# Young Adult Microglial Deletion of C1q Reduces Engulfment of Synapses and Partially Mitigates Cognitive Impairment in an Aggressive Alzheimer's Disease Mouse Model

**DOI:** 10.1002/glia.70189

**Published:** 2026-07-14

**Authors:** Tiffany J. Petrisko, Shu‐Hui Chu, Angela Gomez‐Arboledas, Blossom Zhang, Andrea J. Tenner

**Affiliations:** ^1^ Department of Molecular Biology & Biochemistry University of California, Irvine Irvine California USA; ^2^ Department of Neurobiology and Behavior University of California Irvine Irvine California USA; ^3^ Department of Pathology and Laboratory Medicine University of California, Irvine, School of Medicine Irvine California USA

**Keywords:** Alzheimer's disease, amyloid, C1q, cognition, complement, microglia, synapse

## Abstract

C1q is a multifunctional protein, including its role as the initiating protein of the classical complement cascade. While classical pathway activation is involved in synaptic pruning during nervous system development, it also contributes to inflammation and cognitive decline in Alzheimer's disease (AD). Constitutive genetic C1q deficiency has been shown to reduce glial activation and attenuate neuronal loss in AD mouse models, but the specific contributions of microglial C1q to AD pathology while avoiding deficits during post‐natal development remain unaddressed. To dissect specific role(s) of microglial C1q in AD progression, we crossed the Cx3cr1^CreERT2^ mouse model that deletes C1q from microglia in young adulthood (8 weeks of age) to the aggressive Arctic48 (Arc) amyloidosis mouse model. At 10 months, young adult microglial C1q deletion (Arc C1q^ΔMG^) was associated with improved spatial memory performance, despite unchanged amyloid plaque burden. Furthermore, Arc C1q^ΔMG^ mice exhibited reduced hippocampal C3 protein levels without altering C3 mRNA. No changes were observed in C5aR1, astrocyte GFAP, or microglial Iba1 protein expression. However, Arc C1q^ΔMG^ mice demonstrated region specific reductions in microglial synaptic engulfment, alongside decreased phagolysosome‐associated amyloid in both microglia and astrocytes, and reduced hippocampal amyloid compaction. These findings support a role for C1q in astrocytic C3 induction and the engulfment of both synapses and amyloid. Importantly, young adult microglial C1q inhibition confers cognitive benefits without exacerbating amyloid pathology, suggesting a therapeutic window in which targeting microglial C1q may help preserve synaptic integrity and modulate the neuroinflammatory processes during the later stages of AD.

## Graphical abstract

- Young adult deletion of microglial C1q reduced engulfment of Vglut1+ synapses and mitigated spatial cognitive deficits at 10 months of age in the amyloidopathy Arctic mouse model of AD.
- Neither fibrillar amyloid plaques nor soluble or insoluble Aβ levels in the hippocampus were affected by young adult microglial loss of C1q despite reduced phagocytosis of amyloid by both microglia and astrocytes.
- Loss of microglial C1q reduced C3 levels but did not affect astrocytic GFAP expression in the Arctic model, indicating altered polarization of astrocytes.

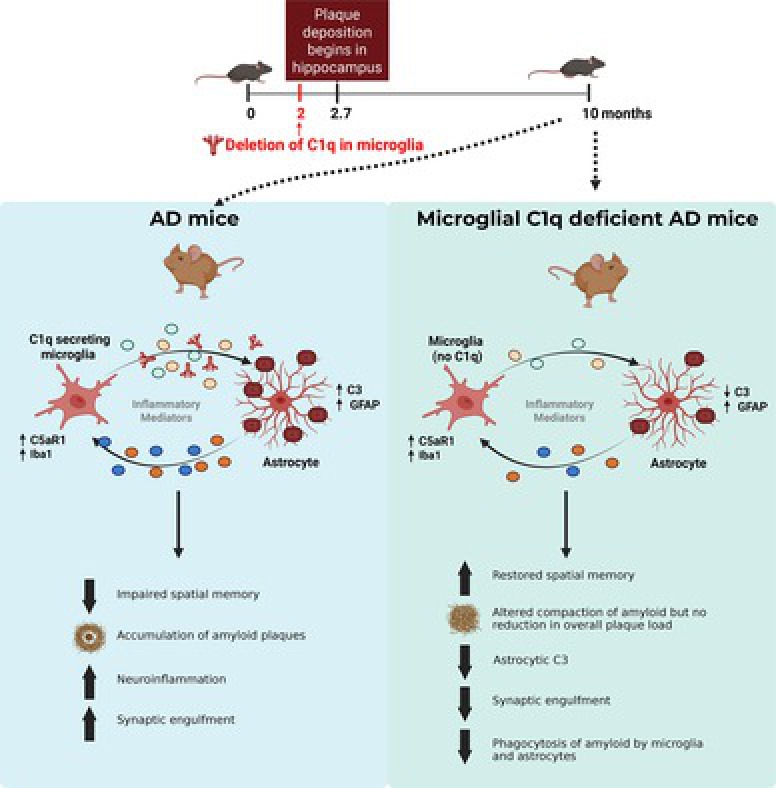

**Abbreviations**
AD: Alzheimer's disease
APP: amyloid precursor protein
Arc: Arctic48
AU: Airy Unit
Aβ: amyloid beta
C1q: complement component 1q
C3: complement component 3
C5aR1: complement component C5a receptor 1
CLU: clusterin
CR1: complement receptor 1
DAMP: damage associated molecular patterns
fAβ: fibrillar amyloid beta
GFAP: glial fibrillary acidic protein
GWAS: genome‐wide association studies
Iba1: ionized calcium‐binding adapter molecular 1
IF: immunofluorescent
Megf10: multiple EGF‐like‐domains 10
MerTK: Mer tyrosine kinase
NfL: neurofilament light
OLM: object location memory
PtdSer: Phosphatidylserine
ThioS: Thioflavin S
TREM2: Triggering Receptor Expressed on Myeloid Cells 2
Vglut1: Vesicular Glutamate Transpoter 1

## 1 Introduction

Alzheimer's disease (AD) is the most common form of dementia in the elderly. In the United States alone, over 7.2 million individuals suffer from AD, with the affected number anticipated to reach 13.8 million by 2060 (Alzheimer's Association 2025). Neuropathologically, AD is characterized by the extracellular accumulation of amyloid‐beta (Aβ) in amyloid plaques and the intracellular accumulation of hyperphosphorylated tau, known as neurofibrillary tangles, as well as synaptic loss (DeTure and Dickson 2019).

Emerging evidence has highlighted the critical role of neuroinflammation and innate immune activation in AD progression (Kinney et al. 2018; Heneka et al. 2025). The complement system, an essential component of the innate immune response, has gained significant attention (Negro‐Demontel et al. 2024; Nimmo et al. 2024; Tenner and Petrisko 2025). Genome‐wide association studies (GWAS) have repeatedly linked several complement components to AD susceptibility, including *CLU* (clusterin) and CR1 (Lambert et al. 2009), and many complement factors colocalize with fAβ plaques in both human and mouse brain tissue (reviewed in Schartz and Tenner (2020)).

Activation of the classical complement pathway begins via engagement of the macromolecular complex C1, comprising the recognition component, C1q, associated with the serine proteases, C1r and C1s. C1 can be activated by C1q binding to the Fc region of IgG or IgM immunoglobulin bound to an antigen or by damage‐ or pathogen‐ associated molecular patterns, apoptotic cells, DNA, or damaged mitochondria. Furthermore, complement activation occurs in response to the pathological hallmarks of AD, namely fibrillar Aβ (fAβ) (Afagh et al. 1996; Velazquez et al. 1997; Tacnet‐Delorme et al. 2001) and hyperphosphorylated tau (Shen et al. 2001).

Classical pathway activation results in the generation of C3a, C3b, and subsequently terminal pathway products C5a and C5b‐9. Previous studies have demonstrated a role for C1q and complement activation in the removal of excess synapses during development (Stevens et al. 2007; Chu et al. 2010; Schafer et al. 2012) and in synaptic plasticity in the adult homeostatic brain (Wang et al. 2020), a process defined as synaptic pruning. C1q expression increases with age, injury, and disease (Stephan et al. 2013; Tenner and Petrisko 2025), and activation of C1 and C3 has also been implicated in AD as a mechanism of synaptic loss and cognitive impairment (Hong et al. 2016; Wu et al. 2019), with constitutive knockouts of C1q restoring synaptic density and reducing pathology in both tauopathy (Dejanovic et al. 2022) and amyloidosis mouse models (Fonseca et al. 2004).

C1q itself is composed of six heterotrimers, each consisting of A, B, and C polypeptide chains, all of which are necessary for proper assembly and function of C1q and contain multiple recognition sites (Sontheimer et al. 2005; Roumenina et al. 2006; Bally et al. 2013). Within the CNS, C1q is produced almost exclusively by microglia (Fonseca et al. 2017; Scott‐Hewitt et al. 2024); however, the contribution of microglial C1q to the progression of AD remains unknown and the use of constitutive knockout models have limited interpretation due to the known developmental and homeostatic roles of C1q. Here, we examined the long‐term effects of microglial C1q deletion during young (8 weeks) adulthood in the aggressive Arctic AD (amyloidosis) mouse model using the Cx3cr1^CreERT2^ knock in mouse, which deletes C1q from microglia by 8 weeks of age independent of tamoxifen treatment (Fonseca et al. 2017). The Arctic mouse, an APP transgenic amyloidosis mouse model that displays early and robust fibrillar amyloid plaque deposition, which contributes to efficient activation of the classical complement cascade (Velazquez et al. 1997; Stoltzner et al. 2000; Tacnet‐Delorme et al. 2001). This model was selected due to its well characterized phenotype, including transcriptomics and behavior, and the effect of complement activation products on those parameters (Hernandez et al. 2017; Schartz et al. 2024; Gomez‐Arboledas et al. 2024). Our results show that deletion of microglial C1q in young adulthood reduces engulfment of hippocampal synapses and mitigates cognitive deficits in 10‐month‐old Arctic mice. Furthermore, while microglial C1q deletion did not affect overall amyloid load at 10 months of age, the structure of amyloid plaques and phagocytosis of amyloid by glia is altered.

## 2 Methods

### 2.1 Animals

All animal experimental procedures were approved by the Institutional Animal Care and Use Committee of the University of California, Irvine, and performed in accordance with the NIH Guide for the Care and Use of Laboratory Animals.

All mice were housed under a 12‐h light/dark cycle with ad libitum access to food and water. Arctic48 (Arc) mice carry the human APP transgene with the Indiana (V717F), Swedish (K670N/M671L), and Arctic (E22G) mutations (under the control of the platelet‐derived growth factor‐ß promoter) on the C57BL/6 background, resulting in the production of amyloid plaques as early as 2–3 months of age (Cheng et al. 2004). To generate microglial specific knockouts of C1q, WT *C1qa* ^*FL/FL*^ and Arc *C1qa* ^*FL/FL*^ mice were crossed to B6.129P2(Cg)‐*Cx3cr1* ^*tm2.1(cre/ERT2)Litt*^/WganJ animals (Jackson, stock #021160) to generate WT *C1qa* ^*FL/FL*^ or Arc C1qa^FL/FL^ *Cx3cr* ^*CreERT2*^ mice (abbreviated C1q^ΔMG^), as previously described (Fonseca et al. 2017). Microglial deletion of C1q in Cx3cr1^CreERT2^ mice was previously shown to occur independently of tamoxifen treatment by 2 months of age (Fonseca et al. 2017). Arctic48 mice were originally obtained from Dr. Lennart Mucke (Gladstone Institute, San Francisco, CA, USA). Both males and females were used in all experiments. WT *C1qa* ^*FL/FL*^ and Arc *C1qa* ^*FL/FL*^ mice, with and without Cx3cr1^CreERT2^ were housed together by sex.

### 2.2 Blood Collection and Plasma Isolation

Blood samples were collected at two time points: 7 and 10 months of age. At 7 months, blood was obtained via submandibular vein puncture using a 5 mm lancet to collect ~100 μL of blood. Pressure was immediately applied to stop bleeding. At 10 months, blood was collected immediately prior to perfusion via cardiac puncture using a 25G syringe. In both cases, blood was collected into EDTA‐containing Eppendorf tubes (10 mM final concentration) maintained on ice. Blood was centrifuged at 2348 g for 10 min at 4°C to separate the plasma. The plasma was immediately aliquoted and stored at −80°C until further analysis.

### 2.3 Tissue Collection

Mice were deeply anesthetized with isoflurane and transcardially perfused with cold phosphate buffered saline (PBS). Brains were rapidly harvested and split along the midline to allow for comparison of the same animal across various biochemical assays. For qPCR, western blot, or Aβ MSD analysis, the hippocampus and cortex were isolated, immediately placed on dry ice, and stored at −80°C. For immunofluorescent analysis, half brains were post‐fixed for 24 h in 4% paraformaldehyde/PBS at 4°C and transferred to 0.02% sodium azide/PBS for long‐term storage at 4°C.

### 2.4 Immunofluorescence (IF)

Brains were cut into 30 μm thick coronal sections on a Leica VT1000S vibratome and stored in 0.02% sodium azide/PBS at 4°C for long‐term storage. Free‐floating sections were washed in 1X PBS. When staining for 6E10, sections underwent antigen retrieval in 50 mM citrate buffer, pH 6.0 for 20 min in 80°C water bath while C5aR1 sections underwent antigen retrieval in 50 mM TBS, 0.05% Tween, pH 9.0 in 80°C water bath for 30 min and allowed to cool for 10 min at room temperature (RT). Sections were then incubated in blocking buffer for 1 h at RT shaking, followed by incubation with primary antibodies in blocking buffer at 4°C overnight (Table 1). Blocking buffer consisted of 5% normal goat serum (NGS), 2% bovine serum albumin (BSA) 0.1% Triton in PBS, except for C1q staining, which used 2% BSA, 0.1% Triton in PBS. Sections were then washed in 1X PBS before secondary antibody incubation (1:500, Table 1) for 1 h at RT, shaking. Sections were then washed in 1XPBS and mounted onto a slide and coverslipped using Liquid Antifade Mounting Medium (Vectashield, Vector) and sealed with nail polish. To visualize plaques, tissues were incubated for 10 min with Thioflavin S (0.5% in MilliQ water, Sigma‐Aldrich #T1892) or AmyloGlo (1:100, Biosensis #TR‐300‐AG in PBS). Within each experiment, 1‐3 sections/mouse were stained and processed at the same time, using batch solutions.

**TABLE 1:** Antibodies for immunofluorescence and western blotting.

Antibody/stain	Host species	Dilution	Antigen retrieval	Supplier‐catalog #	Catalog #	Blocking buffer
Β‐actin	Mouse	1:7000		Sigma	A1978	3% milk
C1q clone 27.1	Rabbit	Hybridoma supernatant		Tenner Lab		2% BSA/0.1% Triton X‐100 in 0.1 M PBS
C1q clone 1151	Rabbit	5.1 μg/mL		Tenner Lab		5% milk
C3 clone 11H9	Rat	1:50		Hycult	HM1045	5% NGS/2% BSA/0.1% Triton X‐100 in 0.1 M PBS
C5aR1 (CD88 10/92)	Rat	1:1000	50 mM TBS, 0.05% Tween, pH 9.0	BioRad	MCA2456	
GFAP	Rabbit	1:2900		Dako	Z0334	
Iba1	Rabbit	1:1000		Wako	019‐19741	
CD68 (clone FA‐11)	Rat	1:1000		BioRad	MCA1957	
Lamp2	Rat	1:1000		Abcam	Ab13524	
6E10	Mouse	1:1000	50 mM citrate buffer, pH 6.0	Biolegend	803001	
Vglut1	Guinea Pig	1:1000		Millipore	AB5905	
Thioflavin S		0.50%		Sigma	T1892	
AmyloGlo		1:100		Biosensis	TR‐300‐AG	
Alexa Fluor IgG (H+L) Goat a Guinea Pig 488	Goat	1:500		Invitrogen	A11073	
Alexa Fluor IgG (H+L) Goat a Mouse 488	Goat	1:500		Invitrogen	A11029	
Alexa Fluor IgG (H+L) Goat a Rat 555	Goat	1:500		Invitrogen	A21434	
Alexa Fluor IgG (H+L) Goat a Rabbit 555	Goat	1:500		Invitrogen	AA21429	
Alexa Fluor IgG (H+L) Goat a Rabbit 647	Goat	1:5000		Invitrogen	A21244	
Peroxidase AffiniPure F(ab′)_2_ fragment Donkey Anti‐Rabbit IgG (H+L)	Donkey	1:5000		Jackson Labs	711‐036‐152	3% or 5% milk
Peroxidase AffiniPure F(ab′)_2_ fragment Donkey Anti‐Mouse IgG (H+L)	Donkey	1:5000		Jackson Labs	715‐036‐150	

### 2.5 Imaging and Quantification

To confirm microglial deletion of C1q within the brain, images of the dentate gyrus were obtained at 10× with the Zeiss Axiovert 200 inverted microscope (Zeiss) and images acquired with a Zeiss Axiocam high‐resolution digital camera (1300 × 1030‐pixel resolution) using ZEN 2.6 software. The intensity of C1q staining was quantified in 4–6 mice/genotype using ImageJ. Five regions of interest (ROI) squares were defined randomly within the molecular layer of the dentate gyrus and the mean pixel intensity per ROI was determined. The mean C1q intensity of each animal was obtained by averaging the intensities of the 5 ROIs and averaged across 1‐2 sections/mouse.

Low magnification images of Thioflavin S (ThioS) staining were obtained using the Zeiss Axio Scan. Z1 slide scanner using a 10× objective. Confocal hippocampal tile scan *z*‐stacks of triple labeled C3/GFAP/Amylo‐Glo and C5aR1/Iba1/Amylo‐Glo were captured using a Leica Sp8 confocal microscope equipped with a 20 × 0.8 numerical aperture (NA) objective using a 1 μm *z*‐step with a 1X zoom. All confocal imaging with the Leica Sp8 microscope utilized a pixel resolution of 1024 × 1024 and pinhole set to 1 Airy Unit (AU), and all settings were maintained within an imaging set for consistency. Using Imaris 9.7 (Oxford Instruments), masks were made of the hippocampus, and surfaces were rendered for each channel, and the percentage of hippocampal surface area (ThioS) or volume (C3, GFAP, C5aR1, Iba1) per channel was calculated. Three hippocampal sections (dorsal, middle, and ventral) were imaged from each mouse and averaged together, with 4–8 mice per genotype.

Images for the engulfment of Vglut1 excitatory synapses by microglia (Iba1/CD68/Vglut1) were obtained with a 63 × 1.4 NA objective with a 0.3 μm step size using a 3.5× zoom. Using Imaris 10.0, synaptic engulfment was quantified in 10–12 microglia cells/mouse per region (CA1 or CA3) with 4–7 mice per genotype, and values were averaged per animal prior to statistical analysis. To quantify microglial and microglial‐phagolysosome engulfment of Vglut1 synaptic puncta, masks were created for Iba1 and CD68 while Vglut1 synaptic puncta were modeled with spots. The number of Vglut1 spots associated with microglia (Iba1) or microglial lysosomes (CD68) was quantified using the Imaris Surface‐Spot Co‐localization feature, using a distance threshold of ≤ 200 nm between Vglut1 and each surface. The proportion of microglia‐ or lysosome‐associated Vglut1 was calculated as the number of Iba1 or CD68 co‐localized Vglut1 puncta divided by the total number of Vglut1 puncta within the same image. To quantify the total volume of Iba1 or CD68 within the *z*‐stack image, the volume of each surface was normalized to the total image volume. All quantitative measures were normalized to the WT mice average in the dataset.

Microglial (Iba1/CD68/6E10) and astrocytic (GFAP/Lamp2/6E10) phagocytosis of amyloid were acquired using a 40 × 1.3 NA objective, with a 0.5 μm step size and a 3.0 zoom. In Imaris 10.0, surfaces were rendered for each channel. Co‐localization masks were created between 6E10 and Iba1 or 6E10 and GFAP to identify amyloid associated with microglia or astrocytes, respectively. The CD68 or Lamp2 signal was then masked and co‐localized within the corresponding 6E10–Iba1 or 6E10–GFAP co‐localization volumes to generate a volume rendering (surface) of phagolysosome‐associated amyloid. The resulting glia‐associated amyloid (6E10–Iba1 or 6E10–GFAP) and lysosome associated amyloid (Iba1‐CD68‐6E10 or GFAP‐Lamp2‐6E10) surface volumes were divided by the total volume of Aβ within the *z*‐stack image. To quantify the total volume of 6E10, Iba1, CD68, GFAP, or Lamp2 expression within the *z*‐stack image, the volume of each surface was normalized to the total image volume. A total of 15‐20 randomly selected hippocampal amyloid plaques per mouse were imaged with values averaged within each animal (4–6 mice per genotype). All quantitative measures were normalized to the mean value of Arc mice.

### 2.6 Western Blot

Hippocampi were pulverized into powder and stored at −80°C. Hippocampi were then solubilized by homogenization in 10 volumes of Tris‐buffered saline (TBS, pH 7.4) containing protease inhibitor cocktail solution (Complete mini, Roche), PhosSTOP (Roche), 1 mM EDTA and 1% sodium dodecyl sulfate (SDS) using a motor pestle for 5 s twice on ice and centrifuged at 18,400 g for 30 min at 4°C. Protein concentration of the supernatant was determined using the BCA protein assay (Pierce, Rockford, IL, USA). 30 μg of hippocampal protein was loaded per lane as previously described (Fonseca et al. 2017). Briefly, samples were subjected to a 10% SDS‐polyacrylamide gel electrophoresis under reducing conditions. Gels were transferred at 4°C in transfer buffer (Tris‐glycine, SDS, and 10% methanol) onto a polyvinylidene difluoride (PVDF, Immobilon‐P, Millipore) membrane at 300 mA for 2 h. The membrane was then blocked for 1 h at RT in 5% nonfat dry milk in TBS‐Tween (TBST), before being incubated with rabbit anti‐mouse C1q (1151) (Huang et al. 1999; Fonseca et al. 2017) in 5% milk overnight at 4°C or β‐actin (Sigma) in 3% milk for 2 h at RT. After 3 washes in TBST for 5 min, membranes were incubated with HRP‐conjugated secondary antibodies diluted to 1:5000 (Jackson Labs) for 1 h at RT. The blots were developed using ECL2 Or ECL (Pierce) and imaged using a BioRad ChemiDoc image system and quantified using Image J software (Fonseca et al. 2004).

### 2.7 qPCR

Total RNA from pulverized mouse brain (10–15 mg) was extracted using the RNeasy Plus Mini Kit (Qiagen). cDNA synthesis was performed using SuperScript III reverse transcriptase (Life Technologies) according to the manufacturer's protocol. Quantitative RT‐PCR was performed using the CFX Duet Real‐Time PCR System and the CFX Maestro software (Bio‐Rad) with the maxima SYBR/Green Master Mix (Thermo Fisher Scientific). The mouse primer sequences for *C3, C4*, *C5ar1* and *Hprt* were obtained from their corresponding reference obtained from Eurofins (Fisher Scientific) (Table 2). cDNA from each hippocampus was tested in triplicate. Within each sample, ΔCt was calculated by subtracting the average of the triplicate cycle threshold (Ct) value of target gene to the average of the triplicate Hprt Ct value. Then the relative expression was calculated by fold difference (2^−ΔCt^) multiplied by 1000.

**TABLE 2:** qPCR primers.

Gene	NCBI ID	Forward	Reverse	Citations
C3	NM_009778.2	GGCCGAGGCACATTGTCGGT	GCTTCTTGGCTGTCTCAGGGGC	(Benoit et al. 2013)
C4	NM_009780 (C4b)	AAACAACCACAACATGCTGC	GTTCTCTGGGAAGGAGGTGC	(Reichwald et al. 2009)
C5aR1		TTCCTGCTGGTGTTCAAG	CTGAGTAGAAGTCCTTATATGC	(Karsten et al. 2015)
HPRT	NM_013556.2	AGCCTAAGATGAGCGCAAGT	ATCAAAAGTCTGGGGACGCA	(Hernandez et al. 2017)

### 2.8 NfL MSD

Plasma NfL was quantified using the MesoScale Discovery R‐Plex Human Neurofilament‐L Assay (cat #: K1517XR‐2) according to the manufacturer's directions. Plasma was diluted 1:1 in Diluent 12. All analyzed samples were within the detection range of the assay and data is expressed in pg/mL plasma.

### 2.9 Object Location Memory

At 38 weeks of age, Cx3cr1^CreERT2^ mice underwent cognitive behavioral testing. Object location memory (OLM) was conducted as previously reported (Hernandez et al. 2017). Briefly, mice were handled for 2–5 min per day for 4 days in the testing room and habituated to the arena (37 × 30 × 23 cm, one wall marked down the center with tape as cue) covered with ~1 cm of sawdust bedding in dim lighting for 5 min a day for 4 days, beginning on the third day of handling. The first day of habituation was recorded as an open‐field trial to measure anxiety and locomotion. During the OLM training sessions, mice were placed into an empty arena for 1 min, placed into individual cages, and 2 identical objects (large blue Legos) were placed across from one another, near the upper wall of the arenas while allowing the mice enough space to pass around the object. Mice were then reintroduced into the arena and allowed to freely explore for 10 min. Mice underwent identical training (objects remaining in same positions) 24 h later. 24 h after this final training session, one object was moved to the opposite corner of the arena (near side wall) to test for object location memory during a 5‐min testing period. After each session, bedding was stirred, and OLM objects were cleaned with 10% ethanol. Different bedding was utilized for males and females, and objects and arenas were cleaned with 70% ethanol and allowed to air dry when switching sexes. All mice were acclimated to room and lighting conditions for a minimum of 1 h each day. Females were always run prior to males. During testing, OLM objects were counterbalanced to control for side preference. All trials were recorded by a mounted camera from above and interactions with objects were scored manually by blinded scorers. The percent discrimination index (%DI) was calculated [((time spent with object moved—time spent with unmoved object)/time spent with both objects) × 100]. Mice were removed from analysis if they spent less than 7 s total with the objects during training, less than 2 s during testing, if they exhibited a preference for one object location over the other during training (±20% DI), or if the performance of the mice was ±2 standard deviations from the mean. Distance traveled, velocity, and time spent in center of arena were analyzed for all habituation sessions using EthoVision V14 (Noldus).

### 2.10 Isolation of Soluble and Insoluble Aβ Fractions

To obtain the soluble and insoluble fractions of Aβ, flash frozen hippocampi were pulverized and 20–30 mg of powder was homogenized in 154 μL of Tissue Protein Extraction Reagent (TPER) with 1 pellet of complete, mini, EDTA‐free Protease Inhibitor Cocktail (Sigma, cat#: 11836170001) and 100 μL Halt Phosphatase Inhibitor Cocktail (Thermo Fisher, cat #: 78426) per 10 mL of TPER. Samples were then centrifuged at 100,000 g for 1 h at 4°C. The supernatant was collected as the soluble Aβ fraction and stored at −80°C. To generate the insoluble fractions, pellets from the TPER‐soluble fractions were homogenized in 75 μL of 70% formic acid. Afterwards, the samples were again centrifuged at 100,000 g for 1 h at 4°C and the supernatant collected as the “insoluble” fraction and stored at −80°C. Formic acid was neutralized with neutralization buffer prior to running MSD (Butler et al. 2024).

### 2.11 Aβ MSD

Hippocampal soluble and insoluble amyloid were quantified using the V‐Plex Aβ Peptide Panel 1 (6E10) kit from MesoScale Discovery (cat: # K15200E) and ran according to manufacturer instructions. Soluble fractions were diluted 1:2 with Diluent 35 while insoluble fractions were diluted to a final concentration of 1:20,000 with Diluent 35 (Butler et al. 2024). Each sample was run in duplicate and normalized to the tissue weight. All samples analyzed were within the detection range of the assay and data are expressed in pg/mg tissue.

### 2.12 Statistical Analysis

All statistical analyses were done with Prism V9.3 (GraphPad). Student's *t*‐test was utilized to compare two groups, while one‐way analysis of variance (ANOVA) followed by Tukey's post hoc test comparing all groups was used to compare WT and Arc genotypes with or without C1q deletion. To compare plasma NfL at 7 and 10 m of age, a two‐way ANOVA with Sidak's post hoc analysis comparing within age (simple effects analysis) was used. Given small and unequal group sizes in some comparisons, results were additionally confirmed using Welch's *t*‐test or Welch's one‐way ANOVA followed by Dunnett's T3 post hoc testing, which yielded identical conclusions. A detailed report of all statistical outcomes presented in the text is available in Supporting Information File S1.

## 3 Results

### 3.1 Young Adult C1q Microglial Deletion Partially Mitigates Cognitive Impairment in Arctic Mice

**FIGURE 1:**
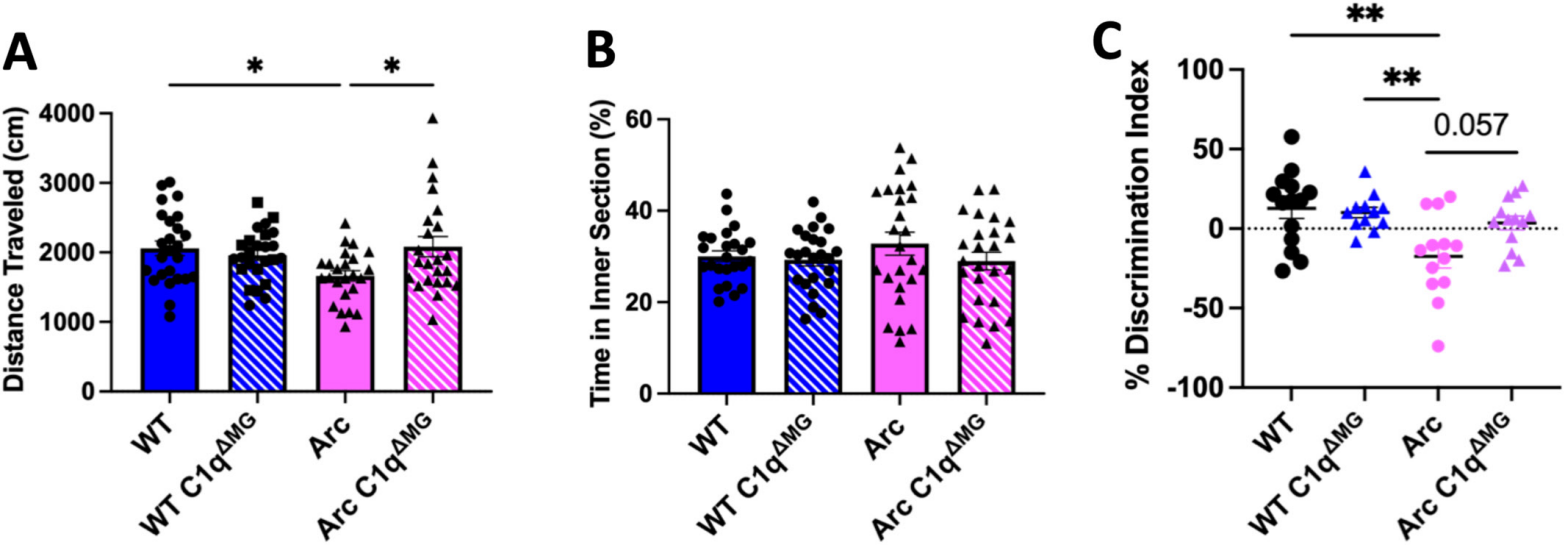
Adult microglial deletion of C1q rescues motor and cognitive impairments in Arctic mice. (A) Behavioral tasks revealed a slight deficit in locomotion in 10‐month‐old Arc mice during 5‐min open field testing (day 1 of habituation to arenas) that was rescued by adult C1q microglial deletion. (B) No changes in anxiety, as measured by the percentage of time spent in the center of the open‐field arena were observed between any groups. (C) Object location memory testing demonstrated a significant impairment in Arc mice compared to WT controls. Deletion of microglial C1q by 8 weeks of age had a near significant (*p* = 0.057) improvement in the cognitive performance in Arc mice at 10‐months of age and eliminated the deficit in Arc mice compared to WT. Each data point represents 1 animal. Data are expressed as Mean ± SEM; *n* = 22–24 per genotype for open field testing and *n* = 13–14 for object location memory. **p < 0.01, ***p < 0.001 by one‐way ANOVA followed by Tukey's post hoc test. Detailed statistical results are provided in Supporting Information File S1.

WT C1qa^FL/FL^ and Arc C1qa^FL/FL^ mice bred to contain Cx3cr1^CreERT2^ (abbreviated WT C1q^ΔMG^ or Arc C1q^ΔMG^) undergo tamoxifen‐independent deletion of C1q at ~8 weeks of age (Fonseca et al. 2017). Microglial deletion of C1q was confirmed in the brain of 10 m old wild type and Arc mice by immunohistochemistry and western blot analysis of the hippocampus (Figure S1A–D). Consistent with a previous report by Fonseca et al. (2017), C1q expression in plasma was not reduced by microglial C1q deletion (Figure S1E,F). In order to assess the functional long‐term consequences of young adult microglial C1q deletion in AD, we assessed behavioral and cognitive performance at 10 months of age. Arc mice displayed a 19% decrease in total distanced moved during five‐minute open field testing compared to WT mice (*p* < 0.05; Figure 1A), which was rescued in Arc C1q^ΔMG^ mice (*p* < 0.05 between Arc‐ Arc C1q^ΔMG^ mice). However, differences in total distance traveled between genotypes were eliminated beginning on Day 2 of habituation. Additionally, no differences in anxiety were noted between groups (Figure 1B), as assessed by the percentage of time spent in the center of the arenas during open field testing. To assess long term hippocampal‐dependent spatial memory, we employed the object location memory (OLM) test (Barker and Warburton 2011; Vogel‐Ciernia and Wood 2014; Chao et al. 2022). No differences in distance traveled or total object interaction time during training between genotypes was observed. 24 h after a 2‐day training period, WT and WT C1q^ΔMG^ animals displayed appropriate preference for the object moved to the novel location. Arc mice demonstrated a significant impairment in spatial memory compared to WT mice (*p* < 0.01; Figure 1C). Arc C1q^ΔMG^ mice showed an attenuation of cognitive deficits compared to Arc controls (i.e., microglial C1q sufficient), with a large effect size (Cohen's *d* = −0.987), although with values approaching statistical significance (*p* = 0.057). In addition, there was no significant difference in OLM performance by Arc C1q^ΔMG^ compared to WT C1q^ΔMG^ (Supporting Information File S1).

### 3.2 Neither Amyloid Pathology nor Plasma NfL Is Affected by Young Adult Microglial Deletion of C1q

**FIGURE 2:**
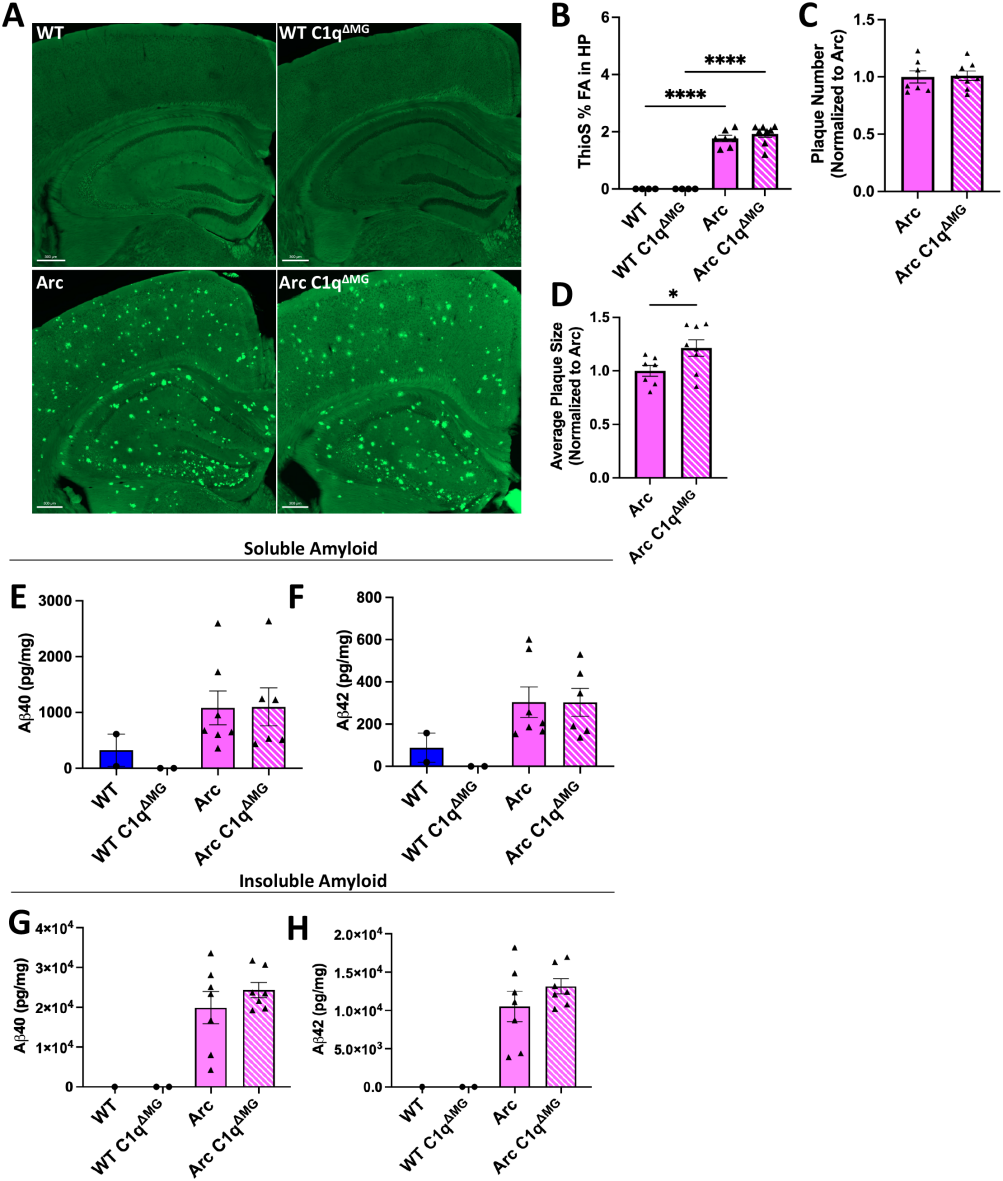
Young adult microglial deletion of C1q fails to alter hippocampal amyloid burden. (A) Representative images of amyloid plaques (Thioflavin S; green) in the hippocampus and cortex. Scale bar: 300 μm. (B–D) Imaris quantification of Thioflavin S field area (B), number of plaques (C), and average plaque size (D) in the hippocampus of WT and Arc mice with and without microglial C1q deletion. Each data point represents the average of 2 sections, *n* = 4–8 mice/genotype and is expressed as Mean ± SEM. Thioflavin S area (B) was analyzed by one‐way ANOVA followed by Tukey's post hoc test. Plaque number (C) and plaque size (area) (D) were analyzed by unpaired *t*‐test. *p < 0.05, ****p < 0.0001. (E–H) Levels of soluble and insoluble Aβ were quantified in dissected hippocampi via MesoScale Multiplex technology. No differences in soluble (E, F) and insoluble (G, H) Aβ_40_ and Aβ_42_ were found in Arc mice following microglial deletion of C1q when analyzed by *t*‐test between Arc and Arc C1q^ΔMG^. Each data point represents 1 animal. Data (E–H) are presented as Mean ± SEM; *n* = 6–7 for Arc genotypes. Detailed statistical results are provided in Supporting Information File S1.

To determine the influence of microglial C1q deficiency on amyloid pathology, we first assessed amyloid plaque burden within the hippocampus with Thioflavin S (ThioS), which binds to the beta‐sheet structure of fibrillar Aβ (fAβ) plaques. No difference was observed in fAβ load (ThioS Field Area %) in Arc C1q^ΔMG^ when compared to Arc mice (Figure 2A,B) at 10 months of age. Despite no change in overall amyloid burden or number (Figure 2C), Arc C1q^ΔMG^ mice displayed a significant (21%) increase in the average overall size of amyloid plaques (*p* < 0.04; Figure 2D). Total hippocampal Aβ_40_ and Aβ_42_ was measured in detergent soluble and insoluble fractions. In concordance with overall hippocampal plaque burden and number, no differences were observed in either soluble (Figure 2E,F) or insoluble (Figure 2G,H) amyloid peptides between Arc C1q^ΔMG^ and Arc mice. These results indicate that while microglial C1q may contribute to the compaction of fibrillar amyloid (Boyett et al. 2003), it does not significantly influence amyloidosis in the Arc mouse model.

Plasma NfL is a common biomarker to assess overall neurodegeneration (Giacomucci et al. 2022). Furthermore, plasma C1q and NfL have been correlated to disease severity in both Huntington's disease (Tassoni et al. 2022) and frontotemporal dementia (van der Ende et al. 2022) and outcome in traumatic brain injury (Butler et al. 2025). Here, plasma NfL levels at 7 and 10 months of age were quantified to determine if the young adult deletion of microglial C1q influenced overall neurodegeneration. When assessed at 7 m of age, Arc mice had a trending increase in plasma NfL compared to WT mice (*p* = 0.06; Figure S2) while plasma NfL levels were not elevated in Arc C1q^ΔMG^ compared to WT C1q^ΔMG^ mice. However, by 10 m of age both Arc and Arc C1q^ΔMG^ animals had elevated plasma NfL compared to their respective controls (Figure S2). This suggests that while microglial C1q deletion may temporarily suppress an increase in plasma NfL, it does not lead to a sustained reduction.

### 3.3 Young Microglial Deletion of C1q Reduces Levels of C3 in Arc Brain, While Microglia Iba1, Astrocyte GFAP and C5aR1 Expression Remained Unaffected

**FIGURE 3:**
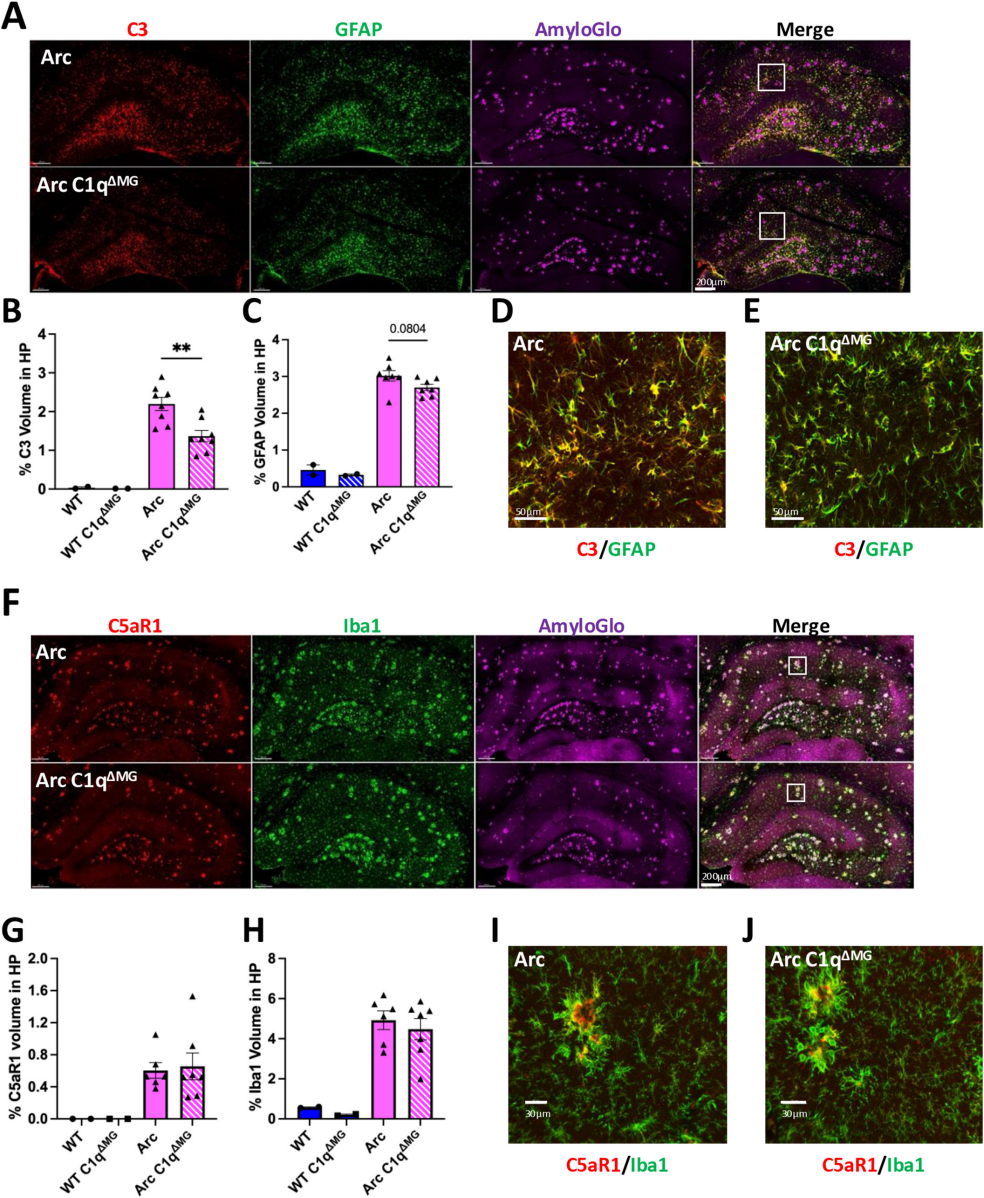
Young microglial deletion of C1q significantly reduces C3 expression but fails to alter glial reactivity or expression of C5aR1. (A) Representative whole hippocampal *z* stacks of C3 (red), GFAP (green), and AmyloGlo (magenta), and merged images at 20× magnification. Scale bar 200 μm. (B, C) Quantification of percent volume of hippocampus of C3 (B), or GFAP (C). (D, E) Representative zoomed region of the 20× confocal image showing merged C3 (red) and GFAP (green), illustrating co‐localization of C3 (yellow) within astrocytes in Arc mice (D) and reduced co‐localization in Arc C1q^ΔMG^ mice (E). Scale bar 50 μm (F) Representative whole hippocampal *z* stacks of C5aR1 (red), Iba1 (green), AmyloGlo (magenta), and merged images at 20× magnification. Scale bar 200 μm. (G, H) Quantification of percent volume of hippocampus of C5aR1 (G), or Iba1 (H). (I, J) Representative zoomed region of the 20× confocal image showing C5aR1 (red) and Iba1 (green), demonstrating similar co‐localization of C5aR1 in microglia in Arc (I) and Arc C1q^ΔMG^ (J) mice. Scale bar 30 μm. Data are shown as Mean ± SEM of the average of 2 sections, with 2 mice for WT genotypes and 6–8 mice for Arc genotypes. *t*‐test between Arc—Arc C1q^ΔMG^.**p < 0.01. Detailed statistical results are provided in Supporting Information File S1.

C3 is known to be elevated in AD patients and AD mouse models (Shi et al. 2015; Wu et al. 2019; Du et al. 2025), including in Arc mice (Carvalho et al. 2022; Schartz et al. 2024). C3 is primarily synthesized by astrocytes in response to inflammatory mediators secreted by microglia, with C1q being identified as a potential key inducer of astrocytic C3 production and induction of neurotoxic astrocytes (Liddelow et al. 2017). To investigate changes in C3 expression and astrocyte reactivity following microglial deletion of C1q, brain sections were stained for C3, GFAP, and Amylo‐Glo. In the hippocampus, Arc mice had an increase in C3 and GFAP expression compared to WT mice (Figures 3A–C and S3A) but significantly reduced C3 reactivity in Arc C1q^ΔMG^ compared to Arc mice (38%; *p* < 0.01; Figure 3A,B,D). Despite this significant reduction in C3 levels, only a trending reduction in GFAP level was observed (10%; *p* = 0.08; Figure 3C,E). Together, these data suggest the loss of microglial C1q is associated with reduced hippocampal C3 levels and is consistent with both a contribution of C1q directly or indirectly to C3 production in astrocytes (Liddelow et al. 2017) and altered complement‐related signaling within the glial environment (Schartz et al. 2024).

Microglia are the primary producers of C1q (Fonseca et al. 2017) and the primary expressors of the complement receptor C5aR1 in AD (Hernandez et al. 2017; Schartz et al. 2024). When activated by its ligand, C5a, C5aR1 activation in microglia promotes inflammatory signaling and chemotaxis of additional immune cells that contribute to AD progression (reviewed in Zelek and Tenner (2025)). To assess the impact of young adult microglial C1q deletion on microglial reactivity and C5aR1 expression, we quantified C5aR1 and Iba1 expression in the hippocampus at 10 months of age. As expected, both C5aR1 and Iba1 expression were increased in Arc mice compared to WT mice (Figure S3B). In Arc mice, microglial C1q deletion did not lead to reductions in either C5aR1 or Iba1 immunoreactivity (Figure 3F–J). These results support previous reports that microglial C5aR1 upregulation occurs as an early response to injury (Carvalho et al. 2022). These data also indicate that microglial C1q is not a major contributor to the hypertrophic morphological state of microglia.

### 3.4 Microglial Deletion of C1q Does Not Reduce Transcription of Select Complement Genes in the Arctic Hippocampus

**FIGURE 4:**
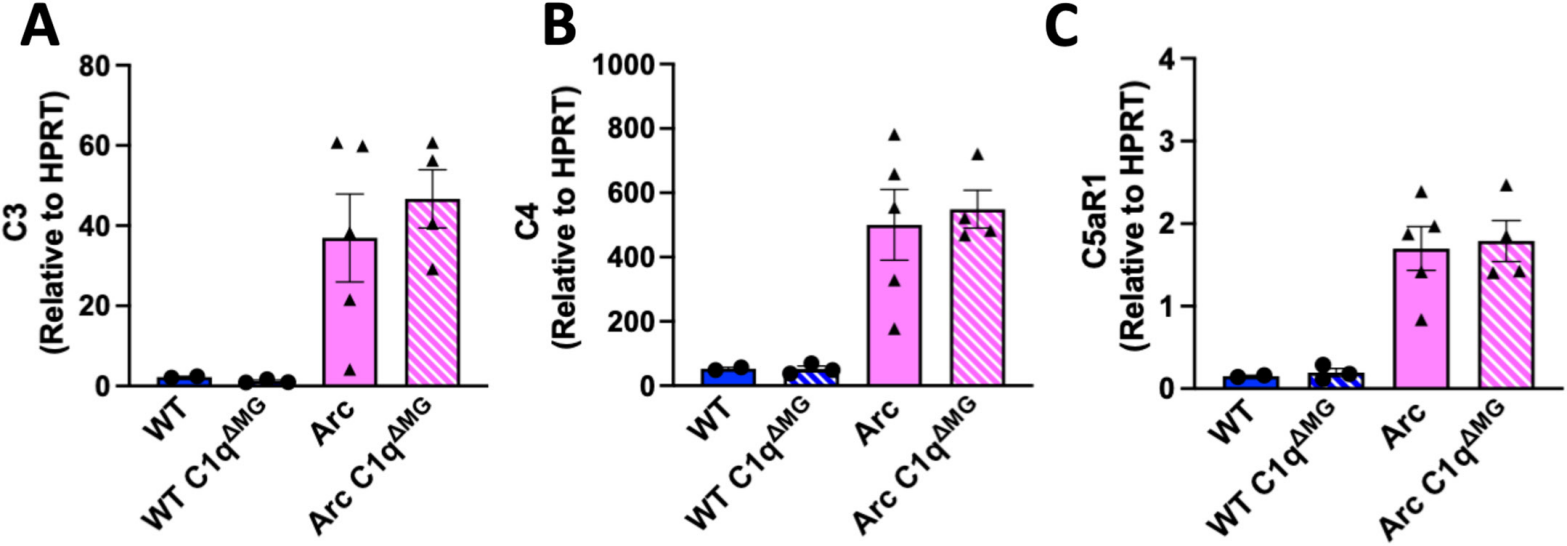
Hippocampal gene expression of C3, C4, and C5aR1 is not altered by loss of microglial C1q. Gene expression of *C3* (A), *C4* (B), and *C5aR1* (C) normalized to *HPRT* expression at 10 months of age. Each data point represents a single animal, run in triplicate. *n* = 2–3 mice for WT genotypes and 4–5 mice for Arc genotypes. Unpaired *t*‐test between Arc—Arc C1q^ΔMG^. Detailed statistical results are provided in Supporting Information File S1.

C4, another component of the classical complement cascade, is induced in the brain in response to injury (Tenner and Petrisko 2025) and its cleavage at the protein level is required to form the classical pathway C3 convertase which participates in synaptic pruning. To determine if reduced C3 gene expression was occurring at the transcriptional level and to determine if loss of microglial C1q influenced mRNA levels of C3, C4, or C5aR1, qPCR for these components was performed. Despite the decrease in C3 protein reactivity in the hippocampus (Figure 3B,E), Arc C1q^ΔMG^ hippocampi had similar C3 gene expression to Arc mice (Figure 4A) at 10 months of age, suggesting that the decrease in C3 expression is occurring at the translational or post‐translational stage. C4 mRNA expression remained elevated in both the Arc and Arc C1q^ΔMG^ hippocampus (Figure 4B) relative to wild type, suggesting its transcription is regulated independently of microglial C1q. Finally, matching our protein expression data (Figure 3E), C5aR1 gene expression is strongly induced in Arc mice but no difference was observed between Arc and Arc C1q^ΔMG^ hippocampi (Figure 4C) at 10 months of age.

### 3.5 Engulfment of Synapses Is Reduced in Arctic Mice Following Young Adult Microglial Deletion of C1q

C1q has a well‐established role in the tagging of synapses for engulfment by microglia both in health and disease (Schafer et al. 2012; Hong et al. 2016; Gyorffy et al. 2018; Kovacs et al. 2020; Gomez‐Arboledas et al. 2024). Here, the impact of microglial C1q on the engulfment of excitatory synapses (Vglut1) by microglia in the CA1 and CA3 subregions of the hippocampus was assessed. These regions were selected as the CA1‐SR and CA3‐SL are known to be part of the tri‐synaptic circuit, which sequentially connects the entorhinal cortex, dentate gyrus, CA3, and CA1 (Basu and Siegelbaum 2015).

**FIGURE 5:**
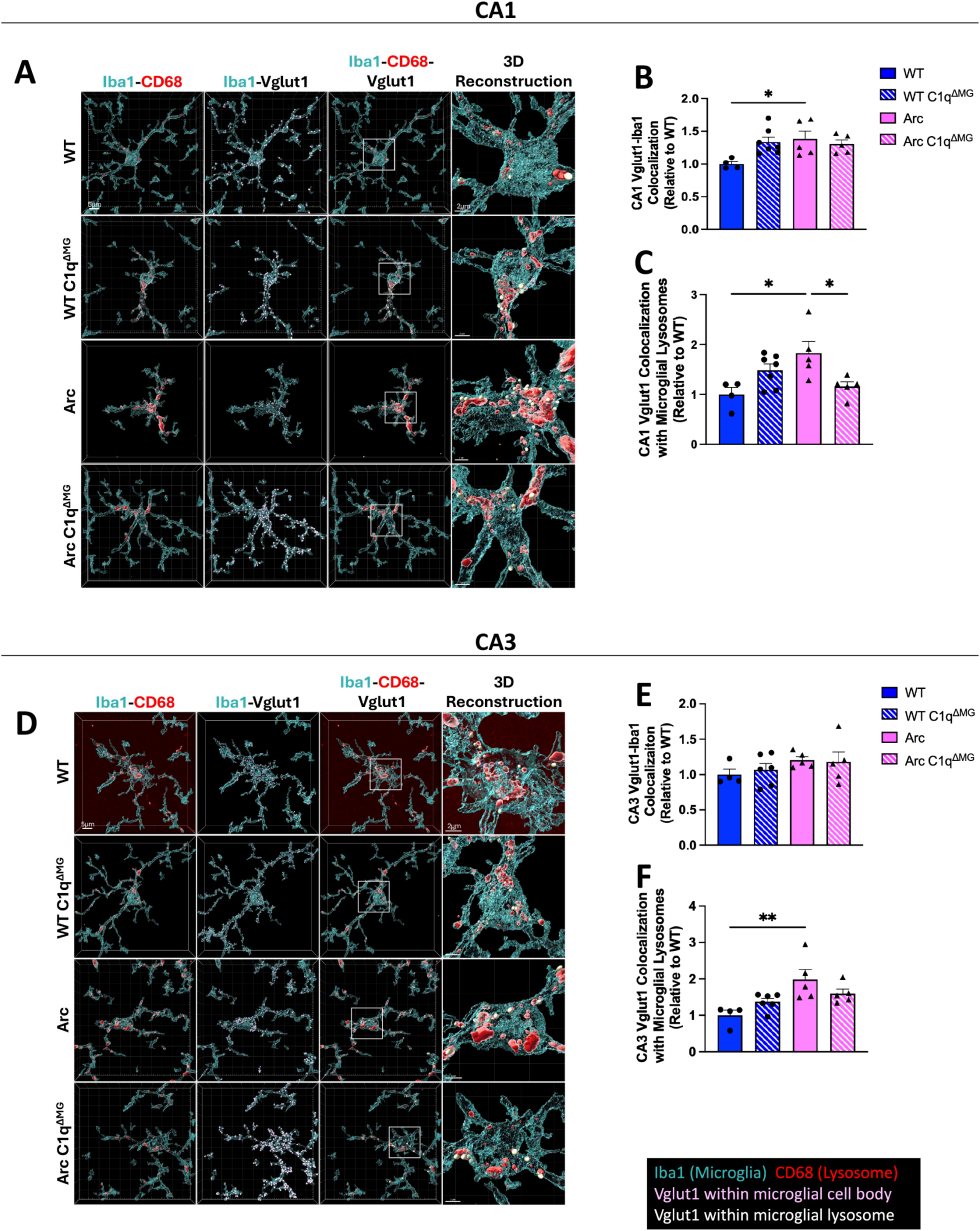
Young microglial deletion of C1q reduced Vglut1 synaptic engulfment in the CA1 but not CA3 subregions of the hippocampus. (A–C) Microglial association and lysosomal localization of Vglut1 in hippocampal CA1. (A) Representative 3D reconstructions of Vglut1 co‐localized with microglia and microglial lysosomes. Iba1 (blue) and CD68 (red) volumes. In the left column panel, the Iba1‐CD68 reconstructions show CD68+ lysosomes (red) within the microglial Iba1 (blue) surface. In the adjacent column, Iba1‐Vglut1 reconstructions demonstrate Vglut1 spots (light purple) within the Iba1 surface (Iba1‐Vglut1 colocalization). The third panel shows Iba1‐CD68‐Vglut1 reconstructions, demonstrating Vglut1 spots (white) localized within CD68+ lysosomes (lysosome‐associated Vglut1). Right most panels show magnified views of the boxed region. (B) Quantitative analysis of Vglut1+ presynaptic puncta localized with microglia in the CA1 region. (C) Quantitative analysis of Vglut1+ presynaptic puncta localized within microglial lysosomes in the CA1. (D–F) Microglial association and lysosomal localization of Vglut1 in hippocampal CA3. (D) Representative 3D reconstructions from CA3 rendered as in panel (A). (E) Quantitative analysis of Vglut1+ presynaptic puncta co‐localized with microglia in the CA3 region (F). Quantitative analysis of Vglut1+ presynaptic puncta localized within microglial lysosomes in the CA3 region. Scale bar: 5, 2 μm insert. Each data point is an individual mouse shown as the average of 10–12 individual microglia cells/mouse per region. *n* = 4–7 mice per genotype, expressed as the ratio of Vglut1 associated with Iba1 (Vglut1 puncta within microglia) or CD68 (microglial lysosome associated Vgut1) relative to total Vglut1 puncta within the image volume, normalized to the WT group mean. Error bars show Mean +/− SEM. *p < 0.05, **p < 0.01, by one‐way ANOVA followed by Tukey's post hoc test. Detailed statistical results are provided in Supporting Information File S1. Representative confocal images, corresponding 3D reconstructions and quantified Iba1and CD68 volumes and number of Vglut1 puncta are shown in Figure S4 (CA1) and Figure S5 (CA3).

In the CA1, but not the CA3, Arc mice had increased engagement of Vglut1 excitatory synapses by microglia (Vglut1‐Iba1 colocalization) compared to WT controls (*p* < 0.05; Figure 5A,B,D,E), although microglial deletion of C1q in Arc animals did not significantly diminish Vglut1‐Iba1 colocalization relative to C1q‐sufficient Arc mice. No change in Iba1 volume was observed in the imaged microglia between genotypes in either the CA1 (Figure S4A,B) or CA3 (Figure S5A,B).

We next assessed lysosomal associated VGlut1. As anticipated, Arc mice demonstrated increased Vglut1 within microglial lysosomes compared to WT mice in both CA1 (*p* < 0.05; Figure 5C) and CA3 regions (*p* < 0.01; Figure 5F). Importantly, the deletion of microglial C1q led to a significant 37% reduction in the amount of Vglut1 present within microglial lysosomes (Vglut1‐Iba1‐CD68) in Arc C1q^ΔMG^ mice compared to normal Arc mice (*p* < 0.05) in the CA1 but not in the CA3 region (19% reduction; Figure 5C,F). While CD68 volume within microglia was not significantly affected by C1q deletion in WT mice (CA1 Figure S4C; CA3 Figure S5C), Arc mice displayed a significant increase in CD68 volume in both regions relative to WT (*p* < 0.01 for CA1 and *p* < 0.05 for CA3). Congruent to the observation that C1q deletion decreased lysosome‐associated Vglut1 in the CA1 (and not CA3) in Arc mice, the volume of CD68 showed a trending 36% reduction in the CA1 (*p* = 0.06; Figure S4C) but not in CA3 (Figure S5C) which may reflect a region‐specific attenuation of microglial activation. Finally, Vglut1 puncta was decreased in the Arc images relative to WT in CA1 (*p* < 0.01) and restored by deletion of microglial C1q (*p* < 0.05) (Figure S4D). While a contribution of basal/homeostatic synaptic turnover cannot be excluded, the presence of significant Arc‐associated Vglut1 loss in CA1, together with its restoration following microglial C1q deletion and the parallel reduction in Vglut1‐CD68 colocalization in Arc C1q^ΔMG^, supports interpretation of the engulfment phenotype as occurring in the context of pathological synaptic vulnerability rather than reflecting only homeostatic pruning. Together, these data suggest that increased microglial synaptic pruning seen at the late (10 m) stage of AD can be partially rescued by young adult microglial deletion of C1q but in a region dependent manner.

### 3.6 Young Adult Deletion of C1q in Microglia Reduces Microglial Response to Plaques, Resulting in Reduced Phagocytosis of Amyloid by Microglia

**FIGURE 6:**
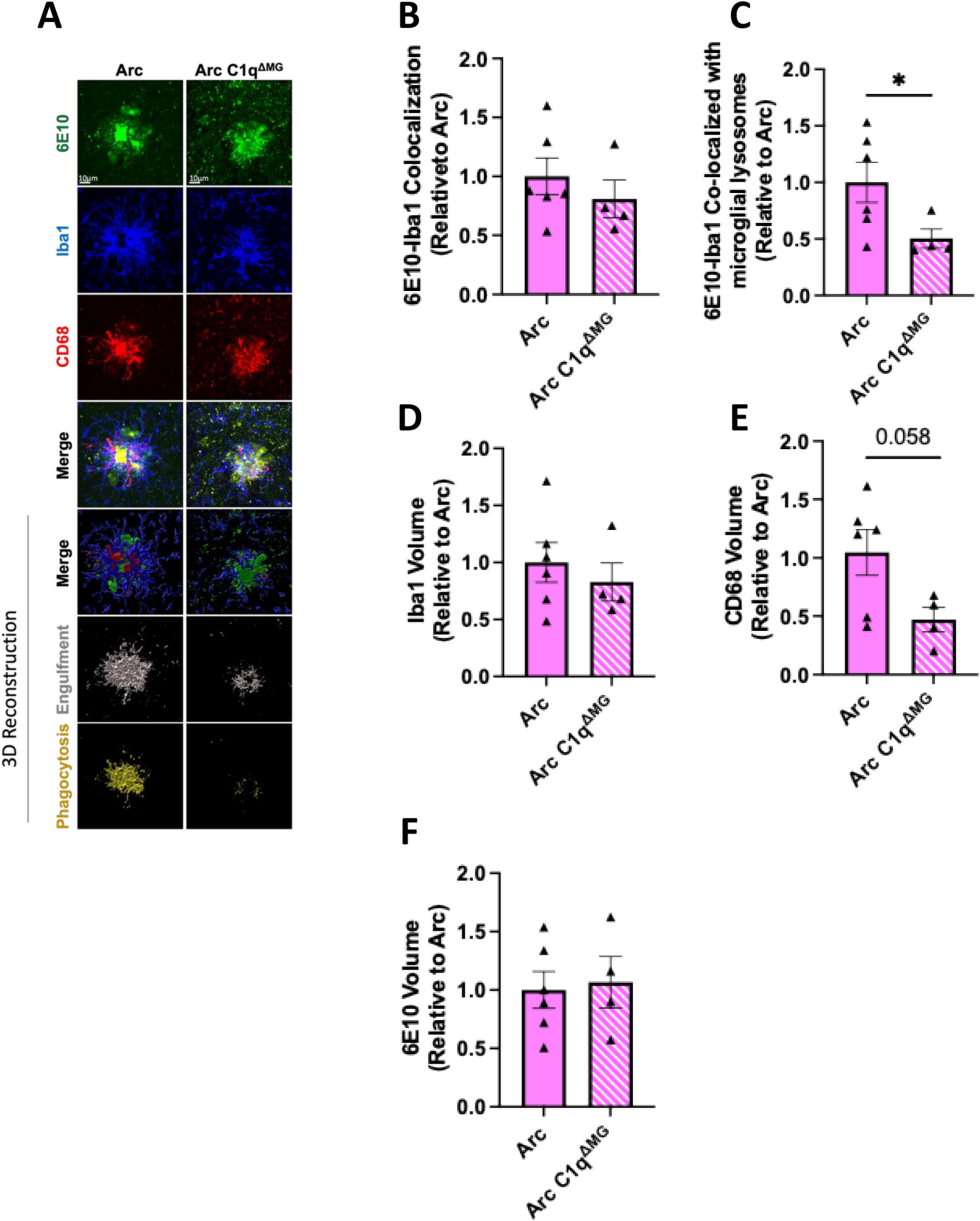
Microglial phagocytosis of amyloid is decreased following young adult microglial deletion of C1q. (A) Representative confocal images and IMARIS 3D renderings of amyloid plaques (6E10; green), microglial cells (Iba1; blue), and microglial lysosomes (CD68; red) in the hippocampus of Arc (left) and Arc C1q^ΔMG^ (right) mice. Scale bar: 10 μm. (B–F) Imaris 3D quantification of internalization of Aβ by microglial cells in Arc C1q^ΔMG^ mice compared to Arc mice (6E10‐Iba1 colocalization) (B) Aβ in microglial lysosomes (6E10‐Iba1‐CD68 colocalization) (C), volume of microglial (Iba1) cells surrounding the amyloid plaques (D), volume of CD68 within field of view (E) and volume of 6E10 Aβ (F). Data points are individual mice with the average of 15–20 hippocampal plaques per mouse, with *n* = 4–6 mice per genotype and normalized to the Arctic genotype. Error bars show Mean +/− SEM. *p < 0.05, as analyzed by unpaired *t*‐test. Detailed statistical results are provided in Supporting Information File S1.

Microglia are known to both aid in the compaction of fAβ and preferentially engulf diffuse Aβ plaques (Condello et al. 2018). To investigate if microglial C1q deletion would alter phagocytosis and/or trafficking of amyloid, the engagement of microglia with amyloid and the amount of Aβ within microglial CD68+ lysosomes surrounding plaques were quantified throughout the Arc and Arc C1q^ΔMG^ hippocampus. Arc C1q^ΔMG^ mice exhibited no change in microglial engagement of amyloid (Iba1‐6E10 colocalization) compared to Arc mice (Figure 6A,B). However, Arc C1q^ΔMG^ mice displayed a significant 50% decrease in the amount of amyloid localized within microglial lysosomes (Iba1‐6E10‐CD68 colocalization) compared to Arc mice (*p* < 0.05, Figure 6C). The reduction in amyloid colocalizing with CD68+ lysosomes in Arc C1q^ΔMG^ mice was independent of Iba1 volume (Figure 6D) and aligns with the observed 53% reduction in CD68 reactivity (*p* = 0.058, Figure 6E). No differences were observed in average volume of 6E10 amyloid per image (Figure 6F), suggesting that these observed changes in CD68 volume are not due to a reduction in amyloid but instead a result of C1q deletion.

### 3.7 Young Adult Deletion of C1q in Microglia Reduces Amyloid Phagocytosis by Astrocytes but Does Not Alter Astrocytic Interactions With Plaques

**FIGURE 7:**
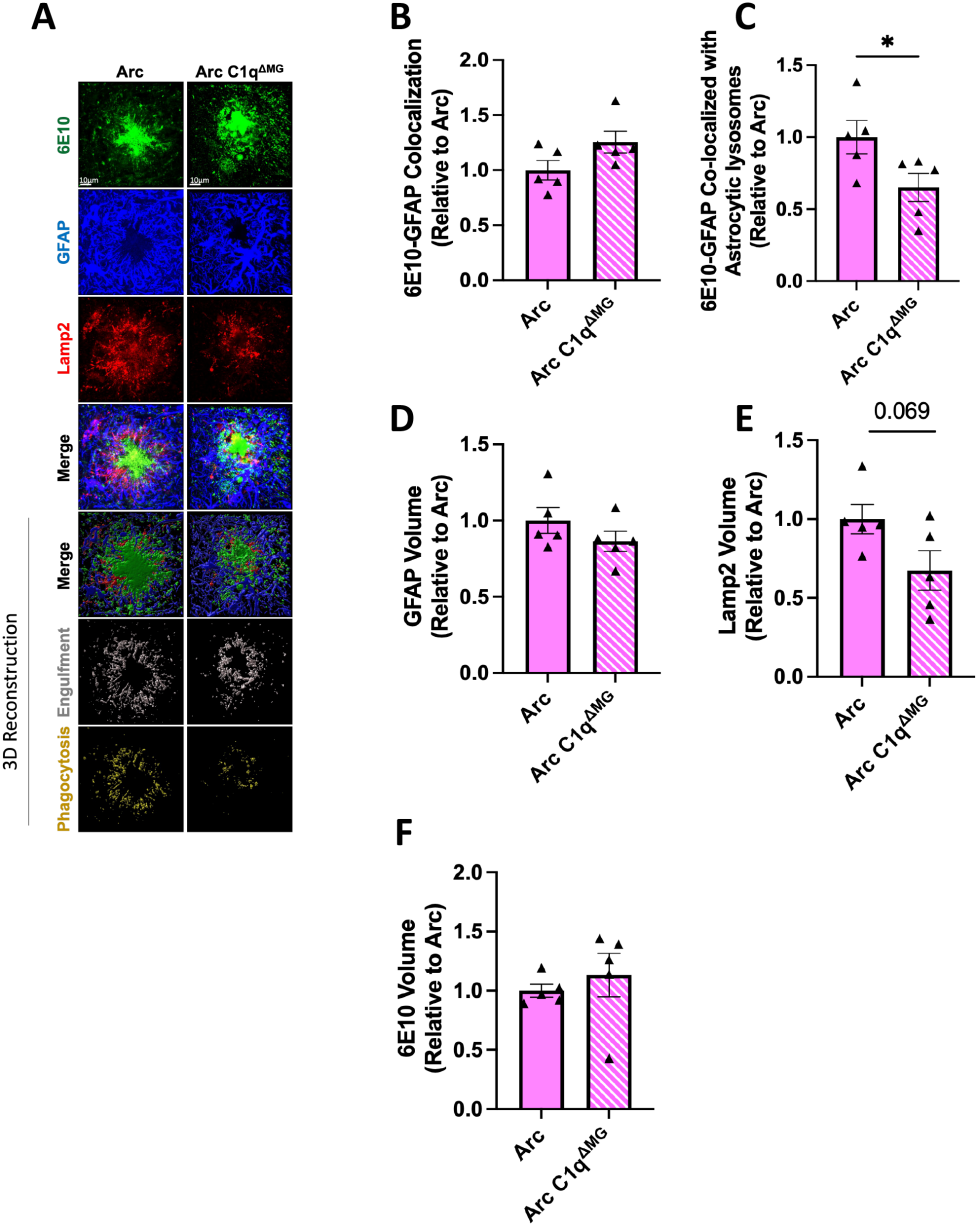
Astrocyte phagocytosis of amyloid is decreased following young adult microglial deletion of C1q. (A) Representative confocal images and IMARIS 3D renderings of amyloid plaques (6E10; green), astrocytes cells (GFAP; blue), and astrocytic lysosomes (Lamp2; red) in the hippocampus of Arc (left) and Arc C1q^ΔMG^ (right) mice. Scale bar: 10 μm. Quantification of Imaris 3D‐volume reconstructions of internalization of Aβ by astrocytes in Arc C1q^ΔMG^ mice compared to Arc mice (6E10‐GFAP colocalization) (B), Aβ in astrocytic lysosomes (6E10‐GFAP‐ Lamp2 colocalization) (C), the volume of astrocytes (GFAP) (D), Lamp2 (E), and 6E10 Aβ within the field of view (F). Data points are individual mice with the average of 15–20 hippocampal plaques per mouse, with *n* = 5 mice per genotype and normalized to Arc genotype. Error bars show Mean +/− SEM. Data was analyzed by unpaired *t*‐test. *p < 0.05. Detailed statistical results are provided in Supporting Information File S1.

Astrocytic association with amyloid (GFAP‐6E10 colocalization) did not differ between Arc and Arc C1q^ΔMG^ mice (Figure 7A,B). Concordant with this data, no change was observed in the overall volume of astrocytes surrounding the plaques (Figure 7D). However, as with microglia, Arc C1q^ΔMG^ mice displayed a significant reduction (35%) in the amount of amyloid located within astrocytic Lamp2+ lysosomes (*p* < 0.05; Figure 7C) when compared to Arc animals. Congruent to this result, Arc C1q^ΔMG^ animals had a trending decrease (33%) in the volume of the lysosome marker, Lamp2 (*p* = 0.069; Figure 7E), surrounding amyloid plaques compared to Arc mice. Once again, the average 6E10‐positive plaque volume was unchanged between Arc and Arc C1q^ΔMG^ mice (Figure 7F). Taken together, this indicates that microglial‐derived C1q contributes to the astrocytic phagocytosis of amyloid.

The observed reduction in lysosomal amyloid by both microglia and astrocytes in Arc C1q^ΔMG^ mice despite unchanged hippocampal fibrillar amyloid burden (Figure 2B) or 6E10 amyloid volume (Figures 6F and 7F) may reflect a broader suppression of neuroinflammatory signaling that may drive APP processing as well as microglial and astrocytic processing or trafficking of amyloid associated with plaques (Cho et al. 2007; Sutinen et al. 2012; Alasmari et al. 2018). Alternatively, the reduction in astrocytic lysosomal amyloid may reflect impaired direct C1q‐mediated astrocytic uptake of amyloid.

## 4 Discussion

The complement system, particularly the classical pathway activated by fibrillar amyloid, hyperphosphorylated tau, and damage associated molecular patterns (DAMPs) binding to C1, plays a critical role in AD pathogenesis through its contributions to synaptic pruning and neuroinflammation (Zelek and Tenner 2025). C1q expression is upregulated as an early response to injury and activates astrocytes to produce C3, enabling excessive synaptic pruning of synapses which can lead to cognitive decline. Microglia are the primary producers of C1q in the brain, and understanding the specific role of microglial‐derived C1q is crucial for elucidating AD mechanisms and developing targeted therapeutics. The present study demonstrates that young adult microglial deletion of C1q in a mouse model of amyloidosis results in improved cognitive function, decreased synaptic pruning, and reduced astrocytic C3 expression despite minimal effects on overall amyloid burden. These results further support the hypothesis that it is the immune response to amyloid accumulation, including activation of a powerful multifaceted complement cascade, rather than amyloid alone that is a driver of synaptic loss and cognitive decline in AD (Hernandez et al. 2017; Miao et al. 2023; Ayyubova and Fazal 2024; Schartz et al. 2024).

Congruent to our results here, a previous study by Fonseca et al. (2004) demonstrated that constitutive genetic C1q deletion in both the Tg2576 and APP/PS1 AD mouse models had no impact on amyloid pathology in aged mice. While synaptic engulfment was not directly assessed in that study, the authors observed a preservation of the synaptic marker synaptophysin as well as the cytoskeletal marker Map2 in aged Tg2576 mice lacking C1q. This result correlates with our observed reduction in Vglut1+ within microglial lysosomes within the CA1 as well as preservation of spatial memory at 10 months of age in Arc C1q^ΔMG^ animals. Furthermore, Fonseca et al. (2004) demonstrated that the deletion of C1q significantly reduced astrocyte and microglia activation by ~50% in both mouse models. In contrast, in the aggressive Arc model our young adult microglial C1q deletion demonstrated no change in either astrocyte GFAP or microglial Iba1 protein expression, reiterating that expression levels of these glial cellular markers alone are not always a reliable surrogate for functional activation states (Schartz et al. 2024).

We observed region‐specific effects of early microglial C1q deletion on microglial synaptic engulfment. Specifically, young adult microglial deletion reduced the amount of Vglut1 inside microglial lysosomes within the CA1 region (Figure 5C), while having minimal impact in the CA3 region (Figure 5F). This differential response may reflect the distinct anatomical and functional properties of these hippocampal subregions within the trisynaptic circuit (Basu and Siegelbaum 2015). Alternatively, in the Arc model, hippocampal amyloid deposition begins around 3 months of age and typically initiates in the dentate gyrus. Given the direct projections from the dentate to CA3, the CA3 may be more vulnerable to early AD pathology, such that C1q deletion in this specific animal model is not early enough to fully mitigate increased synaptic engulfment. Overall, our study demonstrates the importance of assessing both the spatial and temporal role of C1q throughout AD. Indeed, further analysis using spatial transcriptomics and spatial proteomics should provide more mechanistic understanding of these disease processes.

Our observation that microglial C1q deletion led to a 38% reduction in hippocampal C3 levels (Figure 3B) supports previous observations of C1q as a contributor to the induction of astrocytic C3 production (Liddelow et al. 2017; Guttikonda et al. 2021). Although the signaling mechanisms driving C1q‐induced astrocytic C3 production are not fully defined, several studies have demonstrated that astrocytic production of C3 (and subsequent cleavage into C3a which could be independent of C1 (Liszewski et al. 2013)) is critical for mediating the interactions between astrocytes and microglia via C3a‐C3aR signaling (Chen et al. 2020; Wei et al. 2021). While these findings collectively underscore the intricate bidirectional communication between astrocytes and microglia and neuroinflammatory responses, it may also reflect the lack of synergy with downstream complement activation effectors such as C5a and C5b‐9 which would be lacking in the microenvironment of the plaque in microglial C1q‐deleted animals. Notably, although microglial C1q deletion reduced hippocampal C3 protein levels, C3 transcript abundance was unchanged, consistent with post‐transcriptional regulation (Scott‐Hewitt et al. 2024) or altered complement protein stability rather than only reduced transcription. Given that astrocytes are a major source of C3 in the brain, these findings suggest that microglial C1q may indirectly regulate astrocytic C3 processing, cleavage, or turnover, potentially through complement activation dynamics or altered inflammatory signaling.

While overall amyloid load was not altered following young adult microglial C1q deletion, a 21% increase in plaque size was observed, which is compatible with a model in which C1q contributes to amyloid compaction rather than (or in addition to) clearance (Figure 2D). This finding aligns with previous reports that C1q can promote the formation of dense‐core plaques (Boyett et al. 2003). Diffuse plaques have been shown to be more easily internalized by microglia than dense core plaques; however, it has been suggested that this microglial internalization is a mechanism to sequester and compact amyloid into dense core plaques as a means of limiting the effects of amyloid on neurons. Others have shown that the loss of Mer prevented the internalization and conversion of amyloid to a dense core morphology (Huang et al. 2021). Higher levels of diffuse to dense‐core amyloid plaques have also been associated with reduced cognitive impairment (Liu et al. 2022), although this is not consistent in all models (Koffie et al. 2009). While our results support C1q's role in microglial compaction of amyloid (and potentially enhanced fibril formation (Webster et al. 1994)), the observed improvement in cognition with the microglial C1q deletion is likely due to the long‐term absence of downstream complement activation products (C5a and C5b‐9) that induce inflammation and neurotoxicity (Tenner and Petrisko 2025) as well as the contribution of C1q deletion on synaptic pruning.

Glial phagolysosome colocalization of amyloid is also significantly reduced in Arc C1q^ΔMG^ microglia (Figure 6) and astrocytes (Figure 7). This may reflect a general modulation of microglia and astrocytes to a less inflammatory state, as supported by our reduced C3 protein levels, or increased digestion/processing of the amyloid cargo or both. The dynamic interplay between pro‐ and anti‐inflammatory signaling pathways is known to drive amyloid plaque accumulation (Lee et al. 2008; Chouhan et al. 2021). Alternatively, or in addition, the C1q enhanced induction of Lrp1b (Benoit et al. 2013), which reduces amyloid internalization/trafficking/processing, may explain why amyloid levels are unchanged at 10 months of age despite reduced lysosomal colocalization in Arc C1q^ΔMG^ animals. Alternatively, however, the reduction in glial Aβ internalization without altered plaque contact by microglia may suggest that C1q modulates phagocytic efficiency, trafficking or intracellular degradation in addition to plaque compaction. While our data are consistent with a role for C1q in influencing amyloid processing possibly through opsonization, the improvement in cognition likely reflects the sustained reduction in inflammatory signaling (such as C5a‐C5aR1) following microglial C1q deletion. It is also important to note that in this Arc model and some other models, behavior can be rescued in the absence of plaque reduction (Shi et al. 2015; Hernandez et al. 2017).

The molecular mechanisms underlying the observed reduction in lysosomal amyloid in the absence of microglial C1q likely occur through different pathways in microglia and astrocytes. The 50% reduction in microglial lysosomal co‐localized amyloid may reflect the direct role of C1q as an opsonin as well as indirectly due to the generation of C4b/C3b/iC3b facilitating microglial recognition and engulfment of amyloid deposits through complement receptors (Fu et al. 2012; Lv et al. 2024). In contrast, astrocytes express the phagocytic receptors Megf10 (Multiple EGF‐like‐domains 10) and MerTK (Mer tyrosine kinase) (Chung et al. 2013). MerTK facilitates C1q‐mediated phagocytosis, whereas Megf10, whose expression is increased in response to injury, can bind C1q directly. Acting together, MerTK and Megf10 signaling promote astrocytic engulfment of synapses (Chung et al. 2013; Iram et al. 2016; Shi et al. 2021; Zhuang et al. 2023). The 35% reduction in amyloid phagocytosis by astrocytes may be due to disrupted C1q‐Megf10‐MerTK signaling pathways and the general disruption of microglial‐astrocyte communication following C1q deletion may explain impaired clearance function and reduced expression of lysosomal markers in plaque‐associated glia. Alternatively, reduced CD68 and LAMP2 expression may reflect altered glial activation states downstream of C1q loss rather than impaired lysosomal competence per se, consistent with preserved or improved functional outcomes.

The absence of changes in C5aR1 expression following microglial C1q deletion (Figure 3E) is consistent with C5aR1 serving as an early injury response marker that may be regulated independently of C1q signaling (Carvalho et al. 2022; Schartz et al. 2024). However, the absence of C1q reduces the generation of C5a via classical pathway activation, thereby preventing pro‐inflammatory C5aR1 signaling in microglia and further limiting the inflammatory communication between microglia and astrocytes. This supports the hypothesis that other downstream complement activation products (such as C5a and C5b‐9, that would be reduced in the absence of C1q) are indeed the more proximal factors leading to neuroinflammation‐induced cognitive dysfunction (Schartz et al. 2024; Zelek et al. 2025). Although the overall hippocampal volume of Iba1 remained unchanged following C1q deletion (Figure 3F), the observed alterations in both synaptic and amyloid engulfment suggest that microglial gene expression and/or proteome profiles are likely modified despite the preservation of overall microglial morphology and density (Dejanovic et al. 2022; Scott‐Hewitt et al. 2024), consistent with results from other complement genetic deletions (Shi et al. 2015; Hernandez et al. 2017; Carvalho et al. 2022).

This work demonstrates the potential for long‐term cognitive and likely neuroinflammatory benefits from microglial specific C1q deletion beginning in young adulthood in AD. However, several limitations of this study warrant consideration. The Arc mouse model, while valuable for studying amyloid‐related pathology, may not fully recapitulate the complexity of human AD and future studies should utilize newer humanized knock‐in mouse models (Saito et al. 2014; Baglietto‐Vargas et al. 2021; Xia et al. 2022). Furthermore, although we have previously shown that levels of C1q in blood are not altered in this model (Fonseca et al. 2017), because Cx3cr1 can be expressed by other brain‐resident macrophage populations including perivascular macrophages, as well as microglia, it remains possible that C1q production from non‐microglial Cx3CR1 expressing brain macrophages could contribute to detrimental consequences in the C1q sufficient animals. Future analysis that includes spatial transcriptomics should be able to properly address these issues as well as shed light on regional specificity of both microglia polarization and synapse vulnerability. Lastly, future studies incorporating larger cohorts and interaction‐focused statistical designs will help further clarify the contribution of microglial C1q deletion across disease and non‐disease backgrounds.

A study by Dejanovic et al. (2022) demonstrated microglia preferentially engulf inhibitory synapses while astrocytes preferentially engulf excitatory synapses in a C1q‐dependent manner, although astrocytes could compensate for impaired microglial phagocytosis of inhibitory synapses in Trem2 (Triggering Receptor Expressed on Myeloid Cells 2) deficient mice. Our study only examined microglial synaptic engulfment of the excitatory pre‐synaptic protein, Vglut1. Future studies should further investigate the role of microglial C1q deletion on microglial ingestion of inhibitory synapses and on ingestion of both excitatory and inhibitory synapses by astrocytes.

Additionally, future studies should examine the time course of neuroinflammation and cognitive change following microglial C1q deletion. While reduced astrocytic C3 and reduced microglial engulfment of synapses and improved cognition were observed at 10 months of age, in vitro studies have demonstrated that C1q, in the absence of other downstream complement components, as would occur in the earliest stages of disease, is neuroprotective against amyloid‐mediated neuronal cell death (Benoit and Tenner 2011; Benoit et al. 2013). C1q has also been implicated in myelination during development (Yu et al. 2023), as well as promoting repair following spinal cord injuries, indicating that at times, C1q may be beneficial, particularly acutely (Benavente et al. 2020). That some C1q expression can be beneficial is also supported by results from a human clinical trial in which individuals with Guillain Barrè symptoms were treated with the anti‐C1q therapeutic ANX005 (Annexon Biosciences). Patients on the higher, 70 mg/kg dose of ANX005 failed to have an improved outcome at the end of the study, whereas individuals on the lower 35 mg/kg dose showed improved neurological outcomes (Annexon Biosciences 2024). This correlated with levels of anti‐C1q persisting into the recovery phase, suggesting that too much C1q inhibition or inhibiting C1q for long periods may be detrimental.

In summary, this work provides important insights into the temporal dynamics of complement‐mediated neuroinflammation and highlights the potential of targeting C1q in Alzheimer's disease. As previous studies have demonstrated a neuroprotective role for C1q and signaling mechanisms independent of the complement cascade, future studies examining the role of microglial C1q during different stages of AD must be performed. If C1q is indeed neuroprotective in the early stages of AD, anti‐C1q therapeutics could worsen cognitive performance if given too early in disease progression. Finally, understanding the full network of C1q‐responsive receptors across cell types (Benavente et al. 2020) that may mediate C1q functions across disease stages will be essential for developing comprehensive therapeutic strategies targeting C1q.

## Author Contributions

T.J.P. contributed to experimental design, tamoxifen administration, behavioral testing and analysis, tissue collection, IF experiments and analysis, MSD assays, and wrote the manuscript. S.‐H.C. contributed to mice breeding and generation, tamoxifen administration, tissue collection and processing IF experiments and analysis. A.G.‐A. contributed to interpretation of results. B.Z. performed IF and analysis. A.J.T. contributed to experimental design and manuscript preparation. All authors reviewed, edited, and approved the manuscript.

## Funding

This work was supported by NIH R01 AG060148 (A.J.T.), NIH T32 AG000096 (T.J.P.), Alzheimer's Association Research Fellowship 24AARF‐1241864 (T.J.P.), Larry L. Hillblom Foundation postdoctoral fellowship 2021‐A‐020‐FEL (A.G.‐A.), and the Edythe M. Laudati Memorial Fund (A.J.T.). This study was made possible in part through access to the Optical Biology Core Facility of the Developmental Biology Center, a shared resource supported by the Cancer Center Support Grant (NIH CA‐62203) and NIH supported Center for Complex Biological Systems Support Grant (GM‐076516) at the University of California, Irvine.

## Conflicts of Interest

The authors declare no conflicts of interest.

## Supporting information


**Figure S1:** Confirmation of C1q deletion in brain at 10 months of age. (A) Representative images of C1q staining in the dentate gyrus of the dorsal hippocampus. Scale bar represents 100 μm. (B) Quantification of C1q intensity in molecular layer of dentate gyrus. Each data point represents the average mean intensity of 2 sections per animal. *n* = 4–8 mice per genotype. (C) Complete western blot of hippocampal C1q and corresponding β‐actin blot below. The *C1qKO* mouse is a 10‐month Arc C1q gene trapped mouse (Fonseca et al. 2017) while the *Ref* sample is a 10‐month Arc C1qa^FL/FL^ mouse to allow for normalization across multiple blots. (D) Quantification of C1q hippocampal western blot normalized to β‐actin. *n* = 2 per genotype. (E) Representative western blot of 1 μL plasma form 10‐month WT and Arc mice with and without microglial C1q. (F) Quantification of plasma C1q western blots per 1 μL plasma. *n* = 4–6 per genotype. All comparisons were analyzed by unpaired *t*‐test. **p < 0.01, ****p < 0.0001. Detailed statistical results are provided in Supporting Information File S1.

**Figure S2:** Young adult microglial C1q deletion transiently suppresses plasma NfL. Plasma NfL was assessed at 7 and 10 m of age. Results expressed as pg/mL plasma. *n* = 6–14 per genotype per timepoint with samples run in duplicate. Two‐way ANOVA followed by Sidak's post hoc test comparing within age only for NfL. *p < 0.05, ***p < 0.001. Detailed statistical results are provided in Supporting Information File S1.

**Figure S3:** Representative images of WT C3/GFAP and C5aR1/Iba1 hippocampal immunohistochemistry. (A) Representative whole hippocampal *z* stacks of C3 (red), GFAP (green), AmyloGlo (magenta), and merged images at 20× magnification. (B) Representative whole hippocampal *z* stacks of C5aR1 (red), Iba1 (green), AmyloGlo (magenta), and merged images at 20× magnification. Images were generated from tiled *z*‐stack acquisitions spanning entire hippocampus. Scale bar represents 200 μm. [Quantification in Figure 3B,C,G,H. Detailed statistical results are provided in Supporting Information File S1].

**Figure S4:** Representative images of microglial synaptic engulfment and quantification of Iba1 and CD68 volume in the CA1. (A) Representative CA1 confocal images of Vglut1 (green), microglial Iba1 (cyan), and lysosomal marker CD68 (red) and IMARIS 3D rendering of Iba1 (blue) and CD68 (red) surfaces and Vglut1 spots. Scale bar 5 μm. (B‐D) Quantification of Iba1 (B) and CD68 (C) volume and the number of Vglut1 puncta (D) per image normalized to the total image volume. Each data point is an individual mouse shown as the average of 10–12 individual microglia cells/mouse per region. *n* = 4–7 mice per genotype with Iba1 and CD68 volume normalized to their respective WT mean. Data analyzed by one‐way ANOVA followed by Tukey's post hoc test. *p < 0.05, **p < 0.01. Detailed statistical results are provided in Supporting Information File S1.

**Figure S5:** Representative confocal images of microglial synaptic engulfment and quantification of Iba1 and CD68 volume in the CA3. (A) Representative CA3 confocal images of Vglut1 (green), microglial Iba1 (cyan), and lysosomal marker CD68 (red) and IMARIS 3D rendering of Iba1 (blue) and CD68 (red) surfaces and Vglut1 spots. Scale bar 5 μm. (B‐D) Quantification of Iba1 (B) and CD68 volume per image and VGlut1 puncta numberper image normalized to the total image volume. Each data point is an individual mouse shown as the average of 10–12 individual microglia cells/mouse per region. *n* = 4–6 mice per genotype, with Iba1 and CD68 volume normalized to their respective WT group mean. Data analyzed by one‐way ANOVA followed by Tukey's post hoc test. *p < 0.05. Detailed statistical results are provided in Supporting Information File S1.

**Data S1:** glia70189‐sup‐0006‐Supplemental_Data_File_1.xlsx.

## Data Availability

Data available upon request.
