# Supplementary figures and images for: Young Adult Microglial Deletion of C1q Reduces Engulfment of Synapses and Partially Mitigates Cognitive Impairment in an Aggressive Alzheimer's Disease Mouse Model
Source: Glia. 2026 Jul 14;74(9):e70189. doi: 10.1002/glia.70189 (PMC13366034; doi:10.1002/glia.70189)

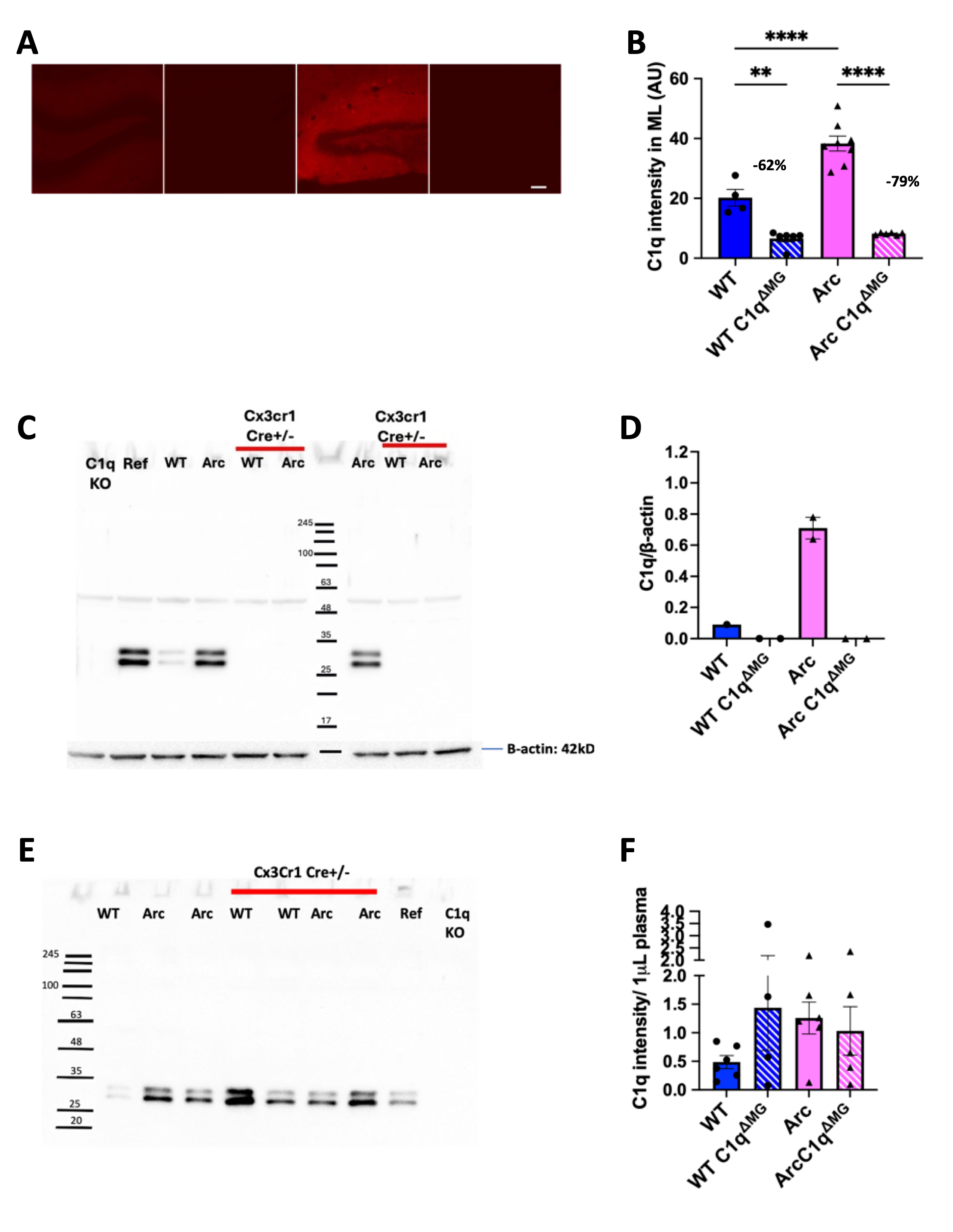

Supplement: Supplementary file 1 — Figure S1: Confirmation of C1q deletion in brain at 10 months of age. (A) Representative images of C1q staining in the dentate gyrus of the dorsal hippocampus. Scale bar represents 100 μm. (B) Quantification of C1q intensity in molecular layer of dentate gyrus. Each data point represents the average mean intensity of 2 sections per animal. n = 4–8 mice per genotype. (C) Complete western blot of hippocampal C1q and corresponding β‐actin blot below. The C1qKO mouse is a 10‐month Arc C1q gene trapped mouse (Fonseca et al. 2017) while the Ref sample is a 10‐month Arc C1qaFL/FL mouse to allow for normalization across multiple blots. (D) Quantification of C1q hippocampal western blot normalized to β‐actin. n = 2 per genotype. (E) Representative western blot of 1 μL plasma form 10‐month WT and Arc mice with and without microglial C1q. (F) Quantification of plasma C1q western blots per 1 μL plasma. n = 4–6 per genotype. All comparisons were analyzed by unpaired t‐test. **p < 0.01, ****p < 0.0001. Detailed statistical results are provided in Supporting Information File S1. [file GLIA-74-0-s003.png]

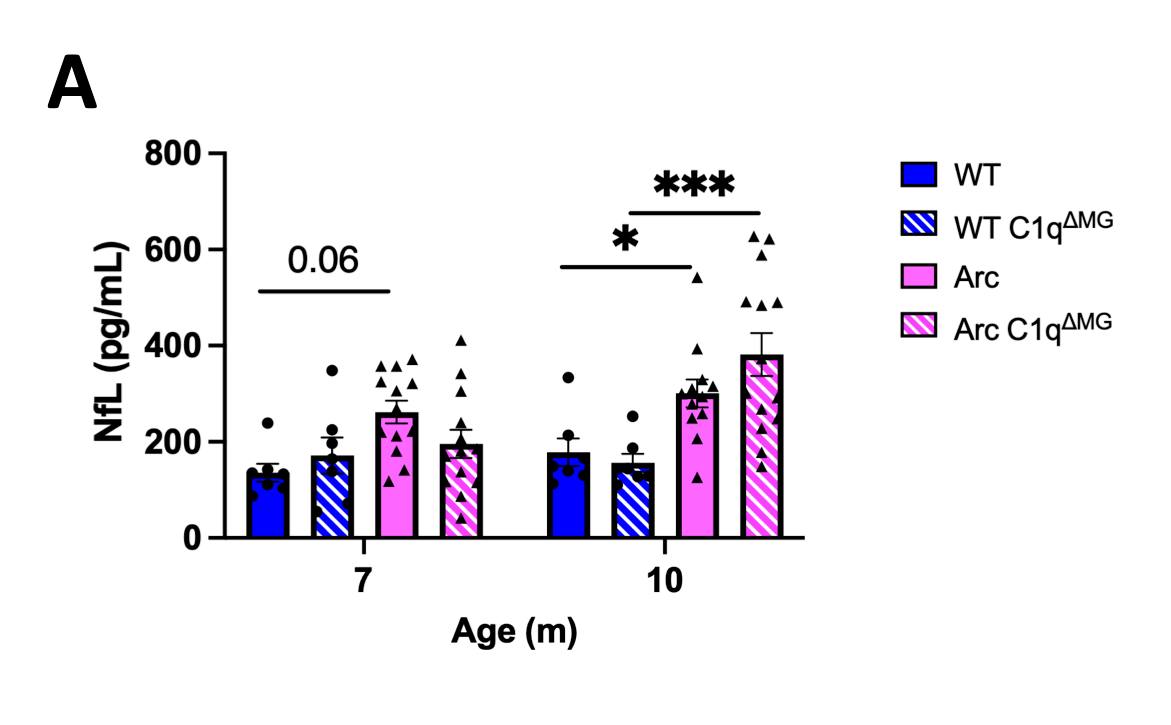

Supplement: Supplementary file 2 — Figure S2: Young adult microglial C1q deletion transiently suppresses plasma NfL. Plasma NfL was assessed at 7 and 10 m of age. Results expressed as pg/mL plasma. n = 6–14 per genotype per timepoint with samples run in duplicate. Two‐way ANOVA followed by Sidak's post hoc test comparing within age only for NfL. *p < 0.05, ***p < 0.001. Detailed statistical results are provided in Supporting Information File S1. [file GLIA-74-0-s002.png]

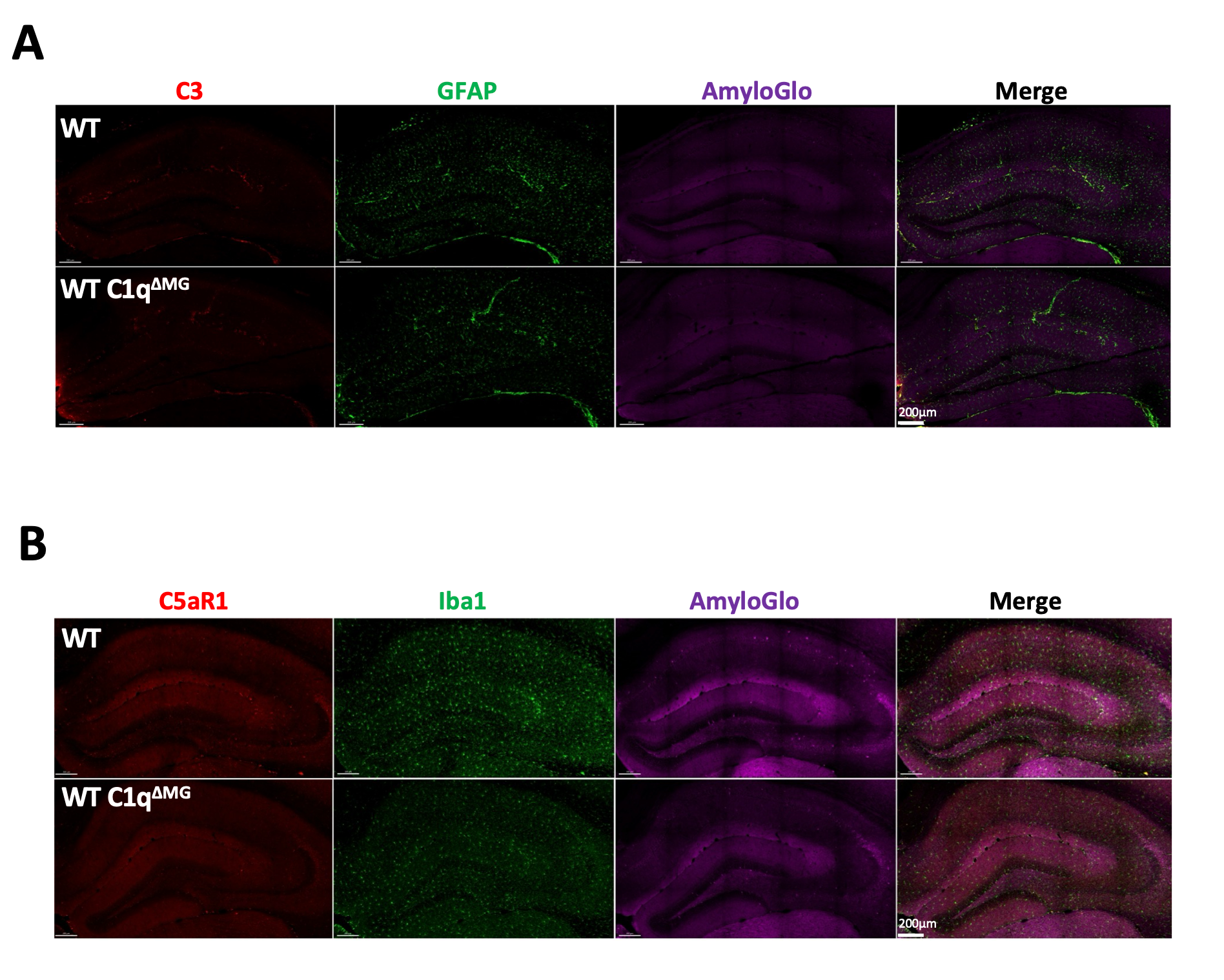

Supplement: Supplementary file 3 — Figure S3: Representative images of WT C3/GFAP and C5aR1/Iba1 hippocampal immunohistochemistry. (A) Representative whole hippocampal z stacks of C3 (red), GFAP (green), AmyloGlo (magenta), and merged images at 20× magnification. (B) Representative whole hippocampal z stacks of C5aR1 (red), Iba1 (green), AmyloGlo (magenta), and merged images at 20× magnification. Images were generated from tiled z‐stack acquisitions spanning entire hippocampus. Scale bar represents 200 μm. [Quantification in Figure 3B,C,G,H. Detailed statistical results are provided in Supporting Information File S1]. [file GLIA-74-0-s005.png]

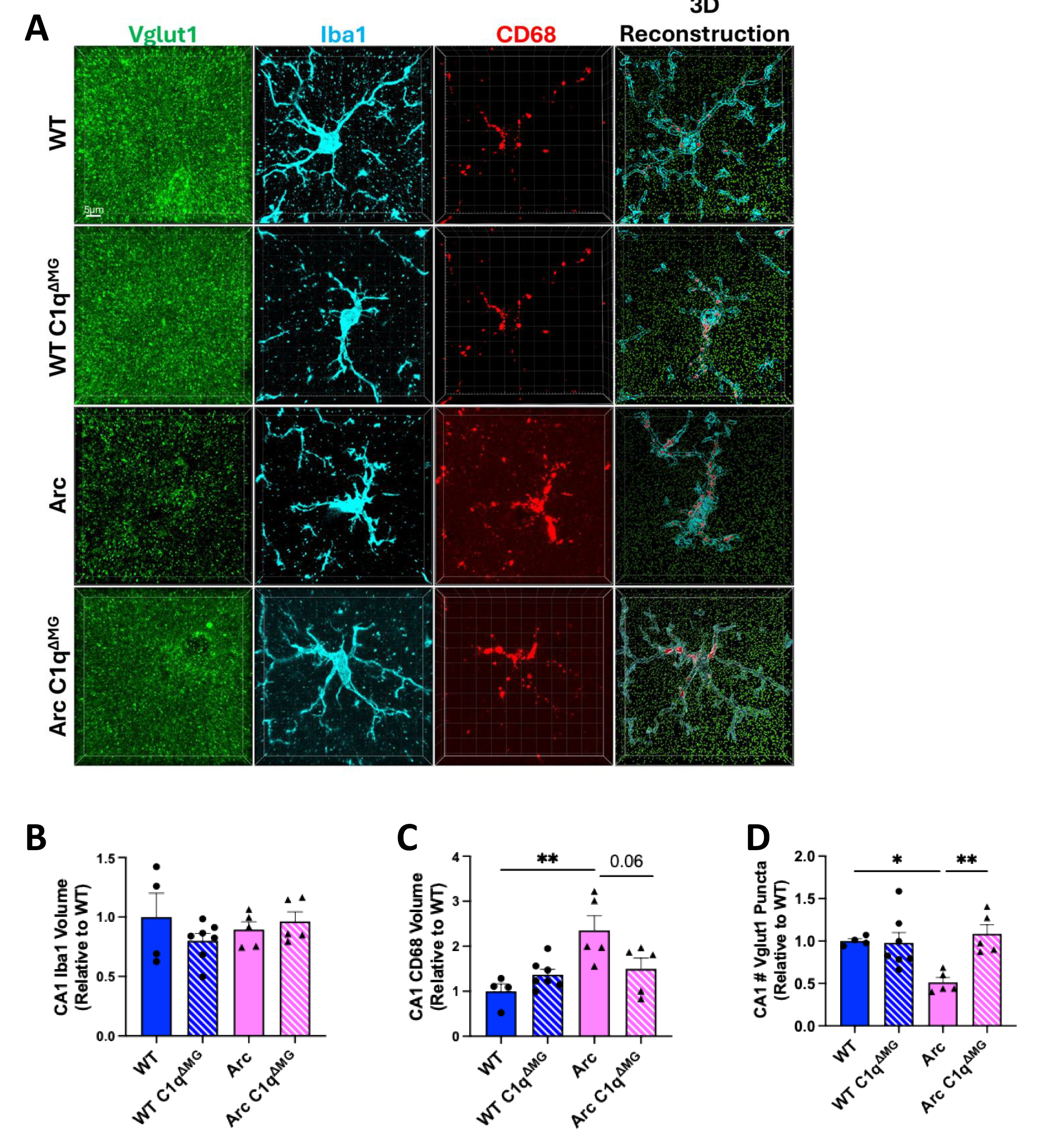

Supplement: Supplementary file 4 — Figure S4: Representative images of microglial synaptic engulfment and quantification of Iba1 and CD68 volume in the CA1. (A) Representative CA1 confocal images of Vglut1 (green), microglial Iba1 (cyan), and lysosomal marker CD68 (red) and IMARIS 3D rendering of Iba1 (blue) and CD68 (red) surfaces and Vglut1 spots. Scale bar 5 μm. (B‐D) Quantification of Iba1 (B) and CD68 (C) volume and the number of Vglut1 puncta (D) per image normalized to the total image volume. Each data point is an individual mouse shown as the average of 10–12 individual microglia cells/mouse per region. n = 4–7 mice per genotype with Iba1 and CD68 volume normalized to their respective WT mean. Data analyzed by one‐way ANOVA followed by Tukey's post hoc test. *p < 0.05, **p < 0.01. Detailed statistical results are provided in Supporting Information File S1. [file GLIA-74-0-s001.png]

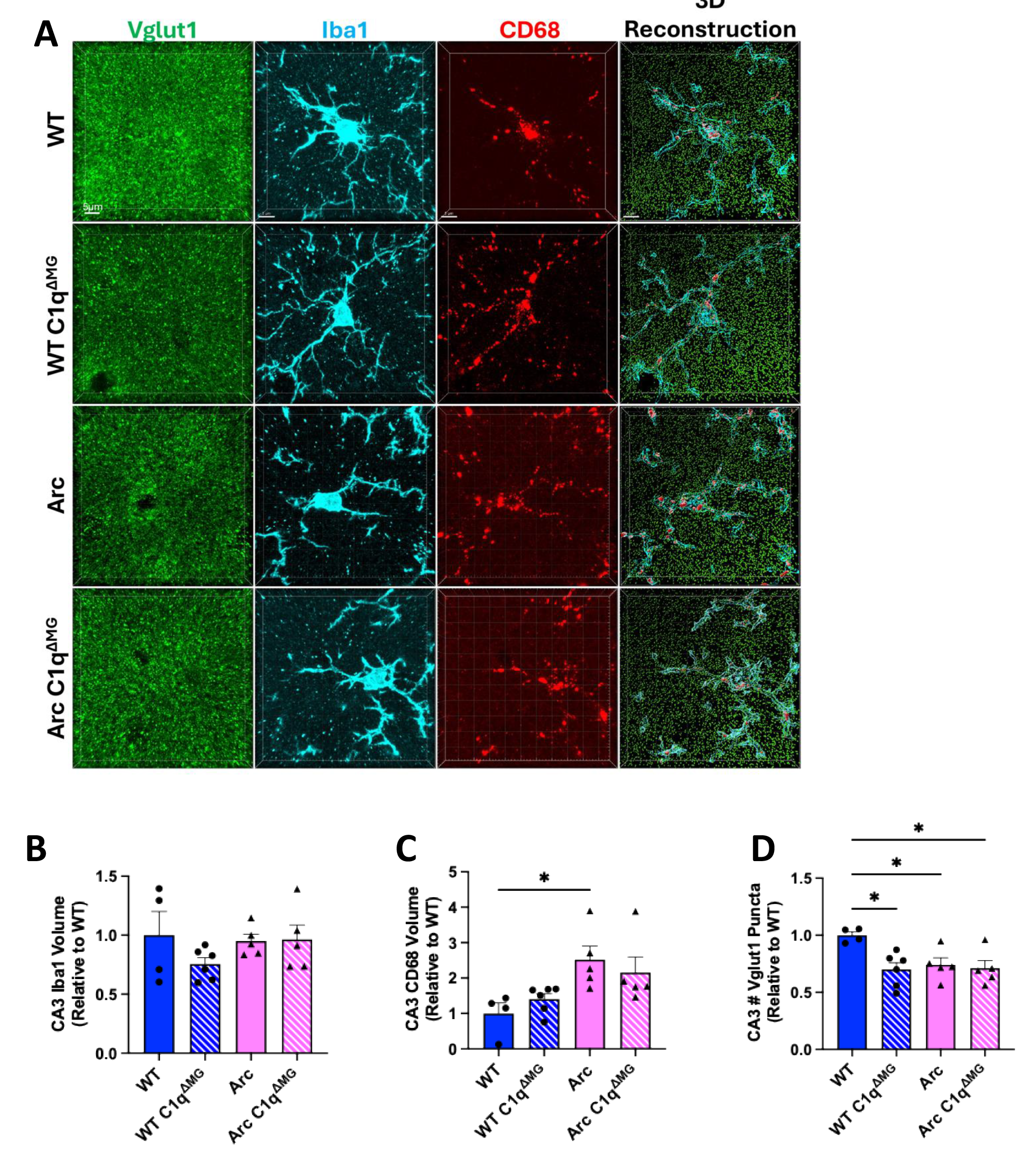

Supplement: Supplementary file 5 — Figure S5: Representative confocal images of microglial synaptic engulfment and quantification of Iba1 and CD68 volume in the CA3. (A) Representative CA3 confocal images of Vglut1 (green), microglial Iba1 (cyan), and lysosomal marker CD68 (red) and IMARIS 3D rendering of Iba1 (blue) and CD68 (red) surfaces and Vglut1 spots. Scale bar 5 μm. (B‐D) Quantification of Iba1 (B) and CD68 volume per image and VGlut1 puncta numberper image normalized to the total image volume. Each data point is an individual mouse shown as the average of 10–12 individual microglia cells/mouse per region. n = 4–6 mice per genotype, with Iba1 and CD68 volume normalized to their respective WT group mean. Data analyzed by one‐way ANOVA followed by Tukey's post hoc test. *p < 0.05. Detailed statistical results are provided in Supporting Information File S1. [file GLIA-74-0-s004.png]
